# Autonomic and neurosensory disorders in dementia with lewy bodies: prevalence and neural basis in the AlphaLewyMA cohort

**DOI:** 10.1186/s13195-025-01935-z

**Published:** 2025-12-19

**Authors:** Morgane Linard, Olivier Bousiges, Mary Mondino, Léa Sanna, Benjamin Cretin, Candice Muller, Pierre Anthony, Catherine Demynck, Nathalie Philippi, Frédéric Blanc

**Affiliations:** 1 ICube Laboratory UMR 7357 and FMTS (Fédération de Médecine Translationnelle de Strasbourg), IMIS team, University of Strasbourg and CNRS, 4 rue Kirschleger, Strasbourg, 67085 France; 2 Laboratory of Biochemistry and Molecular Biology, Hautepierre Hospital, University Hospital of Strasbourg, Strasbourg, 67098 France; 3 Geriatrics Division, CM2R (Research and Resources Memory Centre), Geriatric Day Hospital, University Hospitals of Strasbourg, Strasbourg, 67000 France; 4 Geriatrics Division, CM2R (Research and Resources Memory Centre), Geriatric Day Hospital, General Hospital of Colmar, Colmar, 68024 France

**Keywords:** Dysautonomia, Neurovegetative, Non-motor symptoms, Olfaction, Photophobia, Alpha-synucleinopathy

## Abstract

**Background:**

In dementia with Lewy bodies (DLB), autonomic and neurosensory disorders can precede neurocognitive symptoms by several years. Improving knowledge of these symptoms is essential to avoid associated complications and could reveal potential diagnostic biomarkers and shed light on the pathophysiological mechanisms involved in the early stages of the disease.

**Methods:**

Within the AlphaLewyMA cohort, 142 probable DLB patients at the mild cognitive impairment or dementia stages were screened for 10 autonomic and three neurosensory disorders, at 0, 6, 12, 18, 24, 36, 48, 60, 72, 84 and 96 months of follow-up, using a standardized questionnaire and a test for neurogenic orthostatic hypotension. We described the prevalence and evolution over time of these disorders. To explore their neuroanatomical correlates, we performed whole-brain voxel-based morphometry (VBM) analyses on grey matter volumes in a subsample of 116 patients with MRI data.

**Results:**

The mean age was 71, and 51% were men. Reports of autonomic and neurosensory disorders were very common in our main sample. As some fluctuated during follow-up, repeated screening had a major impact on their prevalence, with 95.7% of the patients declaring an autonomic disorder at least once during the follow-up and 76.8% a neurosensory disorder. The six most frequent symptoms over the follow-up were rhinorrhoea (79.3%), dry mouth (73.3%), sexual dysfunction (70.6%), neurogenic orthostatic hypotension (68.9%), urinary dysfunction (68.0%) and constipation (67.2%). In VBM analysis, dryness (whether ocular, nasal or oral) and severe taste disorders were associated with lower grey matter volumes in the left insula and in the left putamen and caudate nucleus, respectively.

**Conclusion:**

Reports of autonomic and neurosensory disorders were very common and some seemed to fluctuate, highlighting the need for regular, systematic screening. Among these disorders, several symptoms little studied so far in DLB turned out to be the most frequently reported by our patients (rhinorrhoea, dry mouth and sexual dysfunction) and could provide interesting clues for diagnosis. VBM analysis may support an involvement of the insula and certain basal ganglia in dryness-type symptoms and taste disorders. Further prospective studies combining self-reporting and objective measures are needed to refine our results.

**Supplementary Information:**

The online version contains supplementary material available at 10.1186/s13195-025-01935-z.

## Background

Dementia with Lewy bodies (DLB) is a neurodegenerative disease associated with intracellular deposits of alpha-synuclein in the nervous system. As such, it belongs to the family of alpha-synucleinopathies, which also includes Parkinson’s disease (PD) and multi-system atrophy. After Alzheimer’s disease (AD), DLB is considered the second most common cause of neurodegenerative dementia in the elderly, with an estimated prevalence of 15% in autopsy series [1]. Yet, this devastating disease remains largely under-diagnosed [2, 3]. In recent years, criteria have been proposed to improve the diagnosis of DLB at both the dementia and mild cognitive impairment (MCI) stages [4, 5]. These diagnostic criteria include both the progressive onset of cognitive decline and the presence of one or more of the following core clinical features: parkinsonism, fluctuating cognition, rapid eye movement sleep behaviour disorder (RBD) and recurrent visual hallucinations. Two of the four core clinical criteria are sufficient to make a diagnosis of probable DLB.

Beyond these core clinical features, supportive clinical features have also been described, including, among others, neurosensory disorders (NSDs) such as hyposmia and autonomic disorders (AUDs) such as neurogenic orthostatic hypotension (NOH), constipation and urinary dysfunction [5]. Although not specific to DLB, AUDs and NSDs can be useful for the differential diagnosis between DLB and other neurocognitive disorders [5–10], particularly at a prodromal stage. Indeed, AUDs and NSDs are characterized by their onset at an early stage of the disease in DLB, sometimes several years or even decades before the appearance of cognitive impairments [11–13]. Given their early onset, the study of these symptoms could also shed light on the pathophysiological mechanisms involved at an early stage of the disease, including a potential origin of the pathology in the peripheral nervous system [14–17]. Finally, the clinical impact of some of these symptoms is not negligible. For example, cardiac autonomic dysfunction can lead to falls, with a subsequent increased risk of institutionalization and death [18]. Moreover, some AUDs and NSDs can also affect patients’ quality of life, autonomy, cognitive performance and mental health [6, 19–21]. In our experience, failure to recognize such early symptoms as DLB symptoms increases medical wandering and extends patient suffering. In this respect, improving our knowledge of these symptoms is essential so that they can be properly identified and managed [22]. 

Using a cohort of 142 probable DLB patients at the MCI or dementia stages who underwent repeated AUD/NSD screening over a 96-month follow-up, we assessed the prevalence and evolution over time of 13 AUDs/NSDs, including some hitherto little studied. Then, to explore the neuroanatomical correlates of these disorders, we performed whole-brain voxel-based morphometry (VBM) analyses on grey matter (GM) volumes in a subsample of 116 participants with MRI data.

## Methods

### Study design and participants

In the clinical research protocol called AlphaLewyMA [23, 24] (NCT01876459), 242 participants aged 45 and over were enrolled between 2013 and 2016 by experienced neurologists, geriatricians and neuropsychologists from the tertiary Memory Clinic of Alsace in France (Strasbourg and Colmar centres). At baseline, after a detailed clinical evaluation as well as a battery of neuropsychological tests including the Mini-Mental State Examination (MMSE), 121 were diagnosed with probable DLB at the MCI or dementia stages according to McKeith’s 2017 and 2020 criteria [4, 5], 91 were diagnosed with MCI or demented AD according to Dubois’ 2007 criteria [25] and 30 were healthy controls without cognitive complaint recruited among patients’ relatives or from the local clinical investigation centre. Participants were then followed up clinically at 6, 12, 18, 24, 36, 48, 60, 72, 84 and 96 months (except for healthy controls, who were followed up at 24, 48, 72 and 96 months only).

To improve diagnostic accuracy, diagnoses of DLB were reassessed at the end of follow-up (details in Additional file 1). This led to the identification of 142 probable DLB patients at the MCI or dementia stages, constituting our main study sample (flow chart in Additional file 2). According to McKeith’s 2017 and 2020 criteria [4, 5], participants were diagnosed as having probable DLB if they presented a cognitive decline (as evidenced by neuropsychological examination) associated with two or more clinical core criteria among the following: fluctuations, hallucinations, RBD and parkinsonism. The presence of parkinsonism was clinically assessed with the Unified Parkinson’s Disease Rating Scale Part III (UPDRS-III). A questionnaire was offered to the participant and a caregiver (if available) to screen for fluctuations using the Mayo Clinic Fluctuations Scale [26], visual hallucinations using the Parkinson’s disease-associated psychotic symptoms questionnaire [27], and RBD using a questionnaire assessing movements during sleep and vivid dreams and nightmares adapted from Gjerstad et al. [28]. In the absence of Clinical Dementia Rating data, we defined the MCI and dementia groups based on four items from the Instrumental Activities of Daily Living scale (IADL): ability to use the telephone, ability to use transport, responsibility for one’s own treatment, and ability to handle money. The other four items (shopping, food preparation, housekeeping, laundry) were not considered in order to ensure a more consistent assessment between men and women in our sample. Probable DLB patients were considered demented if at least one of these abilities was impaired.

In order to explore the neuroanatomical correlates of AUDs/NSDs in probable DLB patients, we performed whole-brain VBM analyses on GM volumes. Two sub-samples (in Supplementary Fig. 1) were identified, including probable DLB patients with analysable brain MRI data during the follow-up (which could be performed at 0, 24, 48, 72 and/or 96 months) and either.

i) At least one collection of data during the follow-up for each of the following AUDs/NSDs: dry eyes, dry nose, dry mouth, lacrimation, rhinorrhoea, hypersalivation, constipation, smell disorders, taste disorders (*n* = 116 participants).
ii) At least one test for NOH during the follow-up (*n* = 106 participants).

For the first subsample, MRI data were acquired at baseline, 24, 48, 72 and 96 months for 21, 24, 23, 21 and 27 participants, respectively.

All participants provided written informed consent for the study in accordance with the Declaration of Helsinki, and the study was approved by the Ethics Committee of East France (IV).

### Screening for autonomic and neurosensory disorders

Repeated screening for AUD and NSD was carried out at each follow-up visit (0, 6, 12, 18, 24, 36, 48, 60, 72, 84 and 96 months, except for urinary dysfunction, details of which were only collected after 36 months of follow-up) using a standardized questionnaire and a test for NOH.

Regarding AUD, participants were asked to report on the following nine symptoms: dry eyes (absent or present), dry nose (absent or present), dry mouth (absent or present), lacrimation (three categories: absent, occasional or daily/permanent), hypersalivation (three categories: absent, occasional or daily/permanent), rhinorrhoea (three categories: absent, occasional or daily/permanent; excluding seasonal rhinorrhoea), constipation (three categories: absent, occasional but not requiring treatment or frequent or requiring laxatives), urinary dysfunction (any types of urinary symptoms; three categories: no change from their previous state, slight change or severe change) and sexual dysfunction (including change in libido and erectile dysfunction; three categories: no change from their previous state, slight change or severe change).

The presence of NOH was defined as a drop ≥ 20 mmHg of systolic and/or 10 mmHg of diastolic blood pressure within the first 3 min of standing, associated with an absence of reflex tachycardia, defined as an increase in heart rate of less than 10 bpm. Subjects were considered to have severe NOH if they presented a drop ≥ 30 mmHg of systolic blood pressure.

Regarding NSD, the participant was asked to report the presence or absence of the following three symptoms: photophobia (three categories: absent, occasional/with only certain types of light or permanent/with all types of light), smell disorders (three categories: absent, occasional or daily/permanent) and taste disorders (three categories: absent, occasional or daily/permanent).

### MRI acquisition

Using a 3 T MRI scanner (Verio 32-channel Tim Siemens scanner; Siemens), T1-weighted three-dimensional anatomical images were obtained following a volumetric magnetization-prepared rapid acquisition with gradient echo (MPRAGE) sequence (field of view [FOV] = 256 × 256 mm^2^, image matrix = 256 × 256, slice thickness = 1 mm, repetition time = 1900 ms, echo time = 2.52 ms, flip angle = 9°).

### Statistical analysis

#### Prevalence and evolution over time of 13 AUDs/NSDs

The prevalence of each of the 10 AUD and three NSD was described at each follow-up visit separately. The data presented are from the 24- and 36-month follow-ups, as these were the follow-ups with the lowest rates of missing data (due to the absence of AUD/NSD screening in the early years of inclusion in the AlphaLewyMA cohort). For disorders classified into three categories, two variables were constructed: one considering both moderate and severe stages, and one considering only the severe stage. Then, we graphed the evolution of these symptoms over the 11 follow-up visits. To objectively quantify fluctuations over time, we calculated the ratio of the number of visits with a change in the presence/absence of AUDs/NSDs compared with the previous visit to the total number of visits carried out during the follow-up. In view of the fluctuating nature of most symptoms, AUD/NSD prevalences were then calculated over the entire follow-up period. We also estimated the Pearson correlation coefficients between the presence of these symptoms over the entire follow-up period and represented them in a correlation matrix. Multiple testing correction was performed using the Benjamini–Hochberg procedure to control the False Discovery Rate (FDR). Adjusted p-values below 0.05 were considered significant. Analyses were performed with the statistical software SAS (version 9.4; SAS Institute).

#### VBM preprocessing and analyses

VBM preprocessing and statistical analyses were performed using the SPM12 software package (Wellcome Department of Imaging Neuroscience, London, UK; http://www.fil.ion.ucl.ac.uk/) running on Matlab R2024a (MathWorks).

Anatomical MRI images were spatially preprocessed using standard procedures [29]. All T1-weighted structural images were first segmented, bias-corrected, and spatially normalized to the Montreal Neurological Institute (MNI) space using an extension of the unified segmentation procedure that includes six classes of tissue. The DARTEL registration toolbox was then used to build a study-specific template and to bring into alignment all the segmentation images. The VBM analysis was done on modulated GM images; that is, the GM value in each voxel was multiplied by the Jacobian determinant derived from the spatial normalization. This procedure preserves the total amount of GM from the original images. These modulated GM images were smoothed with a Gaussian kernel (full-width at half-maximum [FWHM], 3 mm).

Then, we compared GM volumes between probable DLB patients with or without AUDs/NSDs, adjusting for age and total intracranial volume. VBM analyses were performed using the last MRI performed (which could have been performed at 0, 24, 48, 72 or 96 months). The presence of each AUD/NSD was defined over the follow-up period up to the last MRI performed. When possible, AUDs/NSDs were studied either by combining moderate and severe symptomatology, or by considering only severe symptomatology. Dry eye, dry nose and dry mouth symptoms were also grouped together as a “dryness” variable. Notably, some AUDs/NSDs were not investigated in this neuroimaging study: (i) urinary and sexual dysfunctions, due to higher rates of missing data for these two symptoms; and (ii) photophobia, which has already been studied in a previous article in this sample [30]. No minimal extent of voxels was defined. The results were corrected for multiple comparisons and considered statistically significant for an FDR-corrected *P* < 0.05 at the cluster level. Visualization of results and determination of cluster-related brain regions were carried out using the XjView software package (http://www.alivelearn.net/xjview/).

## Results

### Sociodemographic and clinical characteristics of the study sample

Our study sample consisted of 142 probable DLB patients (84 at the MCI stage and 52 at the dementia stage at baseline; missing data on IADL at inclusion for 6 participants). Their main characteristics are presented in Table 1. Briefly, mean age at baseline was 71.0 ± 9.8 years and 49% were women; mean MMSE at baseline was 25 ± 4 (min 11, max 30). The median follow-up duration was 65 months (1st and 3rd quartiles [Q1-Q3] = 26–96). During follow-up, 43 of the 84 patients with MCI at baseline (51%) progressed to dementia.

**Table 1:** Characteristics of probable dementia with lewy bodies patients

		Probable DLB patients *n* = 142	Patients with MCI at baseline ^b^ *n* = 84	Patients with dementia at baseline *n* = 52
Age at baseline	Mean (SD)	71.0 (9.8)	69.3 (9.4)	74.3 (9.9)
	Min-Max	47.0–91.8	47.0–88.7.0.7	53.0–91.8.0.8
Sex	Female	69 (48.6%)	42 (50.0%)	23 (44.2%)
Educational level (years) ^a^	Mean (SD)	12.1 (4.1)	12.8 (3.9)	11.2 (4.0)
Number of visits	Median [Q1- Q3]	7 [4–9]	8 [5–10]	6 [3–9]
MMSE at baseline ^a^	Mean (SD)	25 (4)	27 (3)	23 (4)
	Min-Max	11–30	17–30	11–30
MMSE at the end of follow up ^a^	Mean (SD)	22 (7)	23 (8)	20 (6)
	Min-Max	0–30	0–30	6–30
Parkinsonism	At least once during follow-up	134 (94.4%)	76 (90.5%)	52 (100%)
Fluctuations ^a^	At least once during follow-up	125 (92.6%)	72 (90.0%)	48 (96.0%)
Visual hallucinations	At least once during follow-up	93 (65.5%)	56 (66.7%)	33 (63.5%)
RBD ^a^	At least once during follow-up	79 (57.7%)	50 (62.5%)	24 (47.1%)
Cholinesterase inhibitors	At least once during follow-up	67 (47.2%)	32 (38.1%)	33 (63.5%)
Antidepressants	At least once during follow-up	78 (54.9%)	48 (57.1%)	26 (50.0%)

*DLB* dementia with Lewy bodies, *MCI* mild cognitive impairment, *MMSE* Mini-Mental StateExamination, *Q1-Q3* 1 st and 3rd quartiles, *RBD* rapid eye movement sleep behaviour disorder, *SD* standard deviation

^a^ There were missing data for the following variables: education (*n*= 2), MMSE at baseline (*n*=5), MMSE at the end of follow-up (*n*= 13), fluctuations (*n*= 7), RBD (*n*= 5). Percentages are calculated on subjects with no missing data for the variable in question

^b^ There were missing data for IADL at inclusion for 6 patients

### Screening for autonomic and neurosensory disorders

**Fig. 1:**
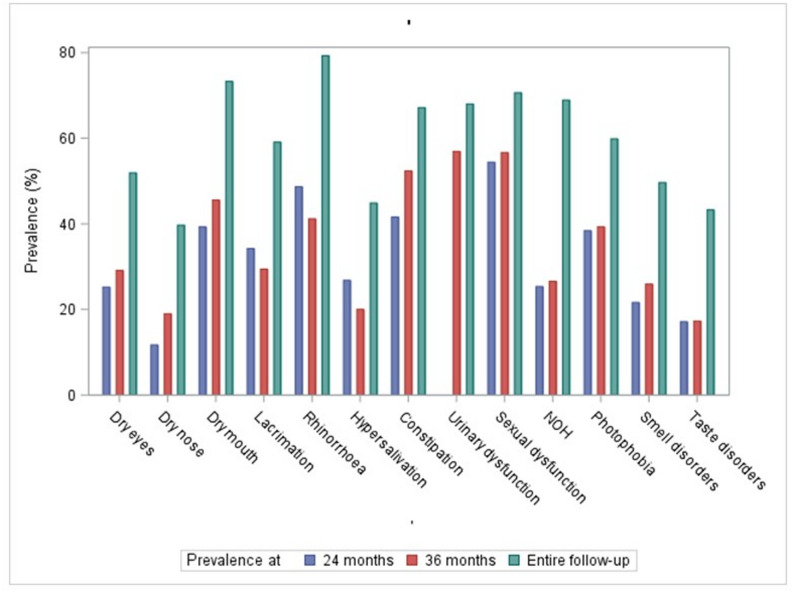
Prevalence of autonomic and neurosensory disorders at 24, 36 months and over the entire follow-up As a reminder, urinary dysfunction was not assessed until the 36-month follow-up. NOH = neurogenic orthostatic hypotension

Firstly, we assessed the prevalence of AUDs/NSDs, considering each follow-up visit separately. As an example, the prevalence at 24 and 36 months of follow-up (i.e. visits with the lowest rates of missing data) are shown in Table 2 and illustrated in Fig. 1. At 36 months, 91.8% of patients reported at least one AUD and 50.6% at least one NSD. The median number of AUD/NSD per participant was 4 (Q1-Q3 = 3–6). The six most frequent symptoms were urinary dysfunction (56.9%), sexual dysfunction (56.7%), constipation (52.4%), dry mouth (45.6%), rhinorrhoea (41.2%) and photophobia (39.3%). Notably, while missing data rates were low for the majority of AUDs/NSDs, this was not the case for sexual dysfunction (30.2% of missing values), urinary dysfunction (24.4%) or NOH (25.6%), which may have led to their prevalence being over- or underestimated. The prevalence of symptoms did not differ significantly between participants in the MCI or dementia stages, with the exception of nasal dryness and photophobia, which were reported less frequently in subjects with dementia (see Additional file 3 for detailed results). At 24 months, the six most frequent AUDs/NSDs were similar, except for urinary dysfunction (not assessed before the 36-month follow-up). The median number of AUD/NSD per participant was 3 (Q1-Q3 = 1–5). Interestingly, the prevalence of each of these symptoms at 24 months was low in the healthy controls included in the AlphaLewyMA cohort (*n* = 15, mean age: 63 ± 8 years, 27% men): the only symptoms with a prevalence over 10% in healthy controls were NOH (20.0%), sexual dysfunction (14.3%) and dry eyes (13.3%) (see Additional file 3 for detailed results).

**Fig. 2:**
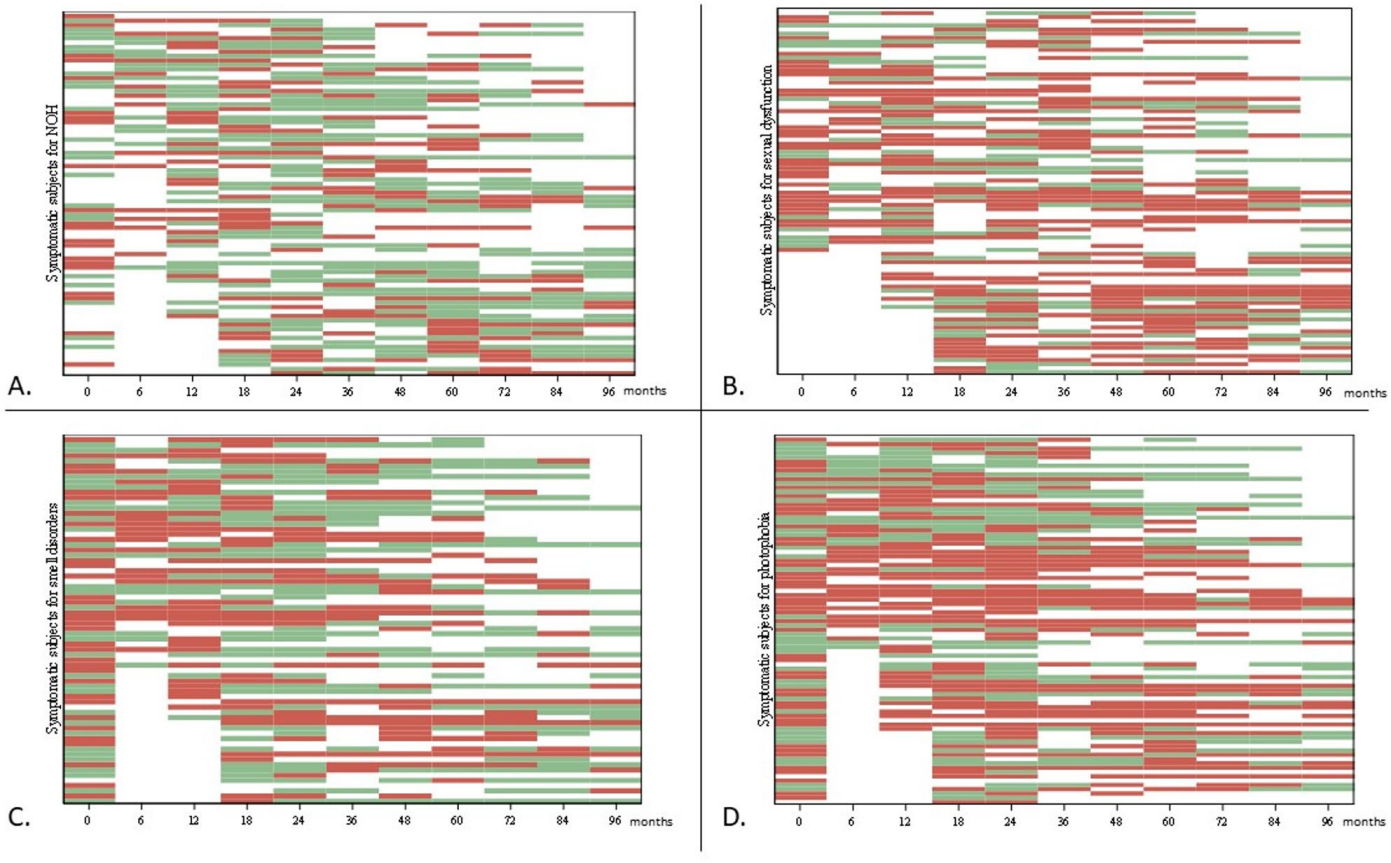
Fluctuations of some autonomic or neurosensory disorders during the entire follow-up Each line represents a subject who was symptomatic at least once in the follow-up for the AUD/NSD considered. Red and green boxes represent follow-up visits associated with the presence (red) or absence (green) of symptoms. Two autonomic disorders (**A**) neurogenic orthostatic hypotension; (**B**) sexual dysfunction) and two neurosensory disorders (**C**) smell disorders; (**D**) photophobia) are shown here, some appearing more fluctuating (neurogenic orthostatic hypotension and smell disorders) and others less so (sexual dysfunction and photophobia). Exhaustive data are available in Additional file 4. AUD = autonomic disorders; NOH = neurogenic orthostatic hypotension; NSD = neurosensory disorders

Secondly, thanks to repeated screening for AUDs/NSDs at up to 11 follow-up visits, we were able to assess the temporal evolution of these symptoms. Visual representations highlighted fluctuations in the presence of most symptoms (see Fig. 2 for some examples and Additional file 4 for exhaustive data). According to the ratio of the number of visits with a change in the presence/absence of each AUD/NSD compared with the previous visit to the total number of visits (see Additional file 4), the most fluctuating symptoms were NOH, dry eyes, dry nose, hypersalivation as well as smell disorders, while urinary dysfunction and sexual dysfunction fluctuated the least.

Consequently, we assessed the prevalence of each AUD/NSD over the entire follow-up (Table 2; Fig. 1), highlighting that 95.7% of patients reported at least once an AUD and 76.8% an NSD. The median number of AUD/NSD per participant was 7 (Q1-Q3 5–10) (see Additional File 3 for a graphical representation over the follow-up). The six most frequent symptoms were consistent with those found at 36 months (except for NOH, which replaced photophobia) but with much higher prevalence: rhinorrhoea (79.3%), dry mouth (73.3%), sexual dysfunction (70.6%), NOH (68.9%), urinary dysfunction (68.0%) and constipation (67.2%). The prevalence of symptoms did not differ significantly between those who took antidepressants during follow-up and those who did not. However, subjects who took a cholinesterase inhibitor during follow-up reported less dry mouth, constipation, photophobia, and smell disorders (see Additional file 3 for detailed results).

**Table 2:** Description of autonomic and neurosensory disorders in 142 probable dementia with lewy bodies patients

	Prevalence at 24 months (*n* = 117)		Prevalence at 36 months (*n* = 86)		Prevalence over the follow-up (*n* = 142)	
	Missing	Present	Missing	Present	Missing	Present
Dry eyes	*6 (5.1%)*	28 (25.2%)	*7 (8.1%)*	23 (29.1%)	*11 (7.8%)*	68 (51.9%)
Dry nose	*6 (5.1%)*	13 (11.7%)	*7 (8.1%)*	15 (19.0%)	*11 (7.8%)*	52 (39.7%)
Dry mouth	*5 (4.3%)*	**44 (39.3%) ④**	*7 (8.1%)*	**36 (45.6%) ④**	*11 (7.8%)*	**96 (73.3%) ②**
Lacrimation	*6 (5.1%)*	**38 (34.2%) ⑥**	*1 (1.2%)*	25 (29.4%)	*10 (7.0%)*	78 (59.1%)
including severe grade		11 (9.9%)		7 (8.2%)		41 (31.1%)
Rhinorrhoea	*4 (3.4%)*	**55 (48.7%) ②**	*1 (1.2%)*	**35 (41.2%) ⑤**	*2 (1.4%)*	**111 (79.3%) ①**
including severe grade		34 (30.1%)		24 (28.2%)		77 (55.0%)
Hypersalivation	*5 (4.3%)*	30 (26.8%)	*1 (1.2%)*	17 (20.0%)	*6 (4.2%)*	61 (44.9%)
including severe grade		10 (8.9%)		7 (8.2%)		31 (22.8%)
Constipation	*4 (3.4%)*	**47 (41.6%) ③**	*2 (2.3%)*	**44 (52.4%) ③**	*5 (3.5%)*	**92 (67.2%) ⑥**
including severe grade		29 (25.7%)		26 (31.0%)		67 (48.9%)
Urinary dysfunction ^a^	*-*	-	*21 (24.4%)*	**37 (56.9%) ①**	*42 (29.6%)*	**68 (68.0%) ⑤**
including severe grade	*-*	-		14 (21.5%)		36 (36.0%)
Sexual dysfunction	*49 (41.9%)*	**37 (54.4%) ①**	*26 (30.2%)*	**34 (56.7%) ②**	*16 (11.3%)*	**89 (70.6%) ③**
including severe grade		29 (42.6%)		24 (40.0%)		78 (61.9%)
NOH	*42 (35.9%)*	19 (25.3%)	*22 (25.6%)*	17 (26.6%)	*23 (16.2%)*	**82 (68.9%) ④**
including severe grade		6 (8.0%)		4 (6.3%)		33 (27.7%)
Photophobia	*5 (4.3%)*	**43 (38.4%) ⑤**	*2 (2.3%)*	**33 (39.3%) ⑥**	*0 (0%)*	85 (59.9%)
including severe grade		19 (17.0%)		15 (17.9%)		46 (32.4%)
Smell disorders	*6 (5.1%)*	24 (21.6%)	*5 (5.8%)*	21 (25.9%)	*1 (0.7%)*	70 (49.6%)
including severe grade		18 (16.2%)		15 (18.5%)		48 (34.0%)
Taste disorders	*6 (5.1%)*	19 (17.1%)	*5 (5.8%)*	14 (17.3%)	*1 (0.7%)*	61 (43.3%)
including severe grade		11 (9.9%)		8 (9.9%)		42 (29.8%)

^a^Urinary dysfunction was not screened until 36 months of follow-up

The six most frequent symptoms are indicated by circles containing the numbers 1 to 6

*NOH* neurogenic orthostatic hypotension

**Fig. 3:**
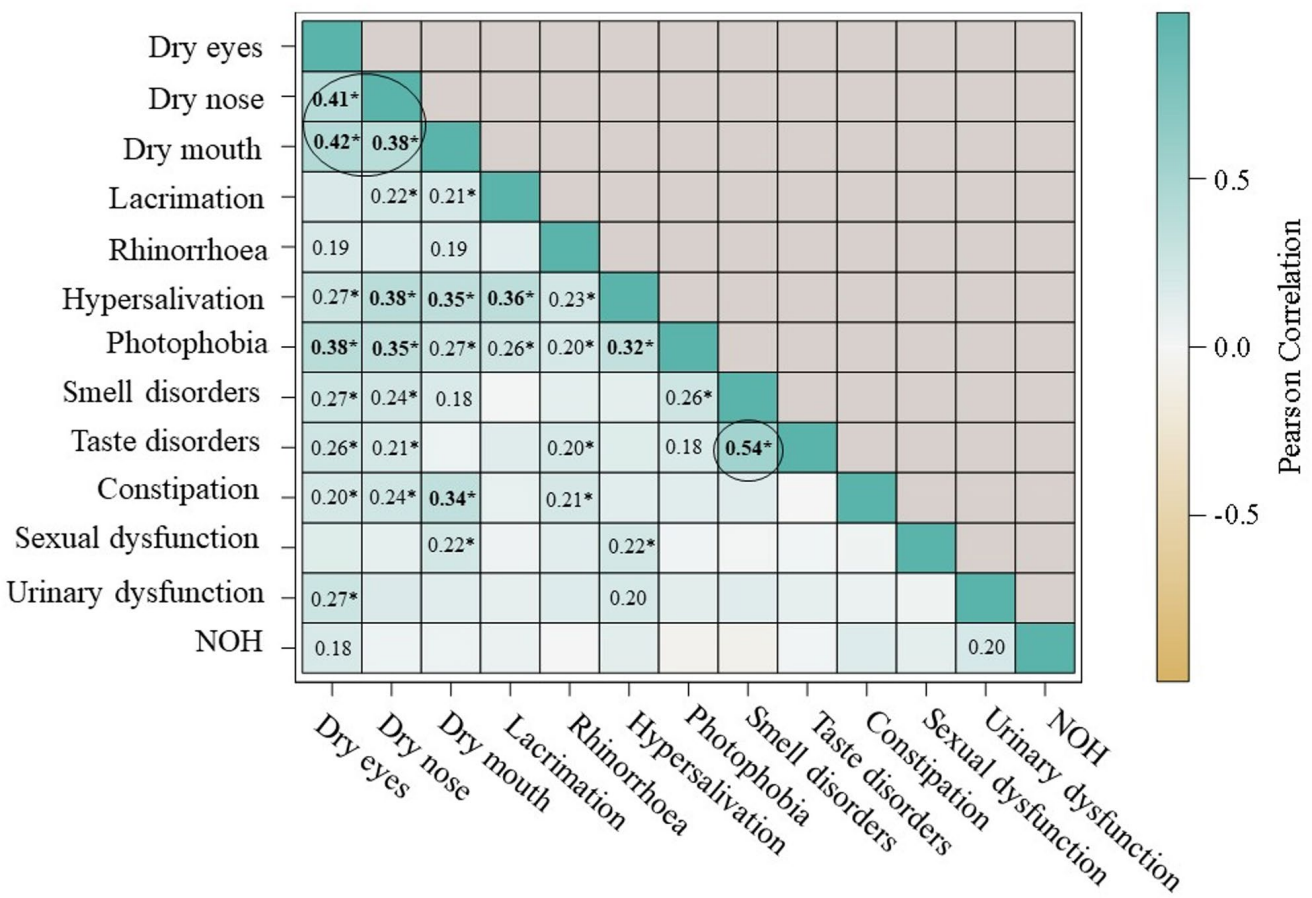
Correlation matrix between each autonomic or neurosensory disorder over the entire follow-up Pearson correlation coefficient values are shown in the matrix only for those associated with a *P* < 0.05. Values marked with an asterisk are those considered statistically significant for an FDR-corrected *P* < 0.05. Values in bold are correlation coefficients > 0.30. AUD = autonomic disorders; DLB = dementia with Lewy bodies; NSD = neurosensory disorders; NOH = neurogenic orthostatic hypotension

Then, we estimated the Pearson correlation coefficients between the presence of each AUD/NSD over the entire follow-up (correlation matrix in Fig. 3). Only low to moderate correlations were observed. The most correlated symptoms were taste and smell disorders (*r* = 0.54), followed by dryness-type symptoms (*r* between 0.38 and 0.42 for ocular, nasal and oral dryness). Hypersalivation was also moderately correlated with dry nose, dry mouth and lacrimation (*r* between 0.35 and 0.38) and photophobia with dry eyes and dry nose (*r* between 0.35 and 0.38). Overall, there seemed to be a modest correlation between the various upper-body symptoms (dryness, hypersecretion, photophobia and, to a lesser extent, taste disorders and smell disorders), while urinary dysfunction, sexual dysfunction and NOH seemed to have little correlation with other symptoms. Notably, there was no negative correlation between dryness and hypersecretion symptoms or between sympathetic and parasympathetic symptoms.

### Neuroimaging results

**Fig. 4:**
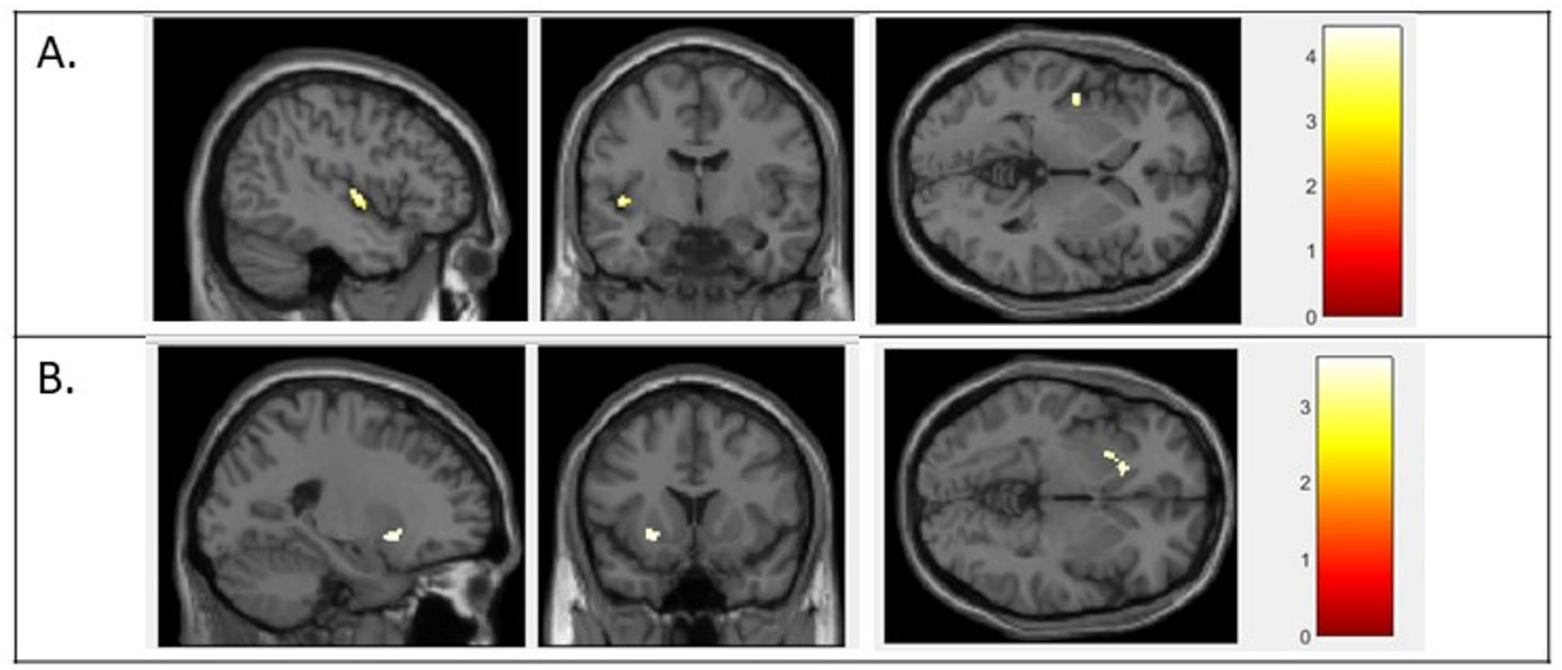
VBM analyses comparing patients with or without (**A**) dryness or (**B**) severe taste disorders After adjustment for age and total intracranial volumes, dryness (either ocular, nasal or oral) was associated with lower grey matter volumes in the left insula and severe taste disorders were associated with lower grey matter volumes in the left caudate nucleus and the left putamen. The results were corrected for multiple comparisons and considered statistically significant for an FDR-corrected *P* < 0.05 at the cluster level. Sagittal, axial and coronal views. FDR = false discovery rate; VBM = voxel-based morphometry

**Fig. 5:**
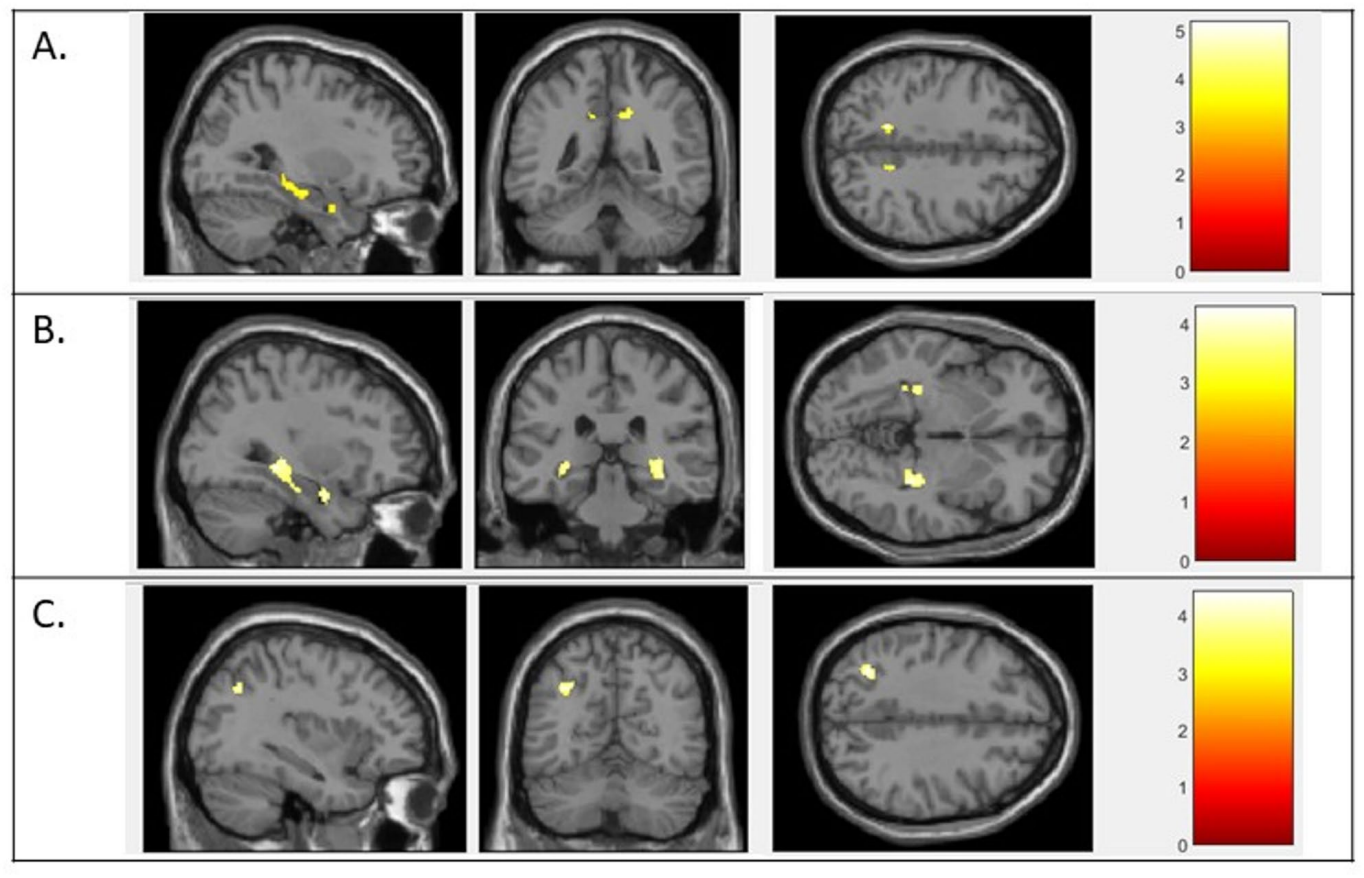
VBM analyses comparing patients with or without (**A**) severe lacrimation, (**B**) hypersalivation and (**C**) severe smell disorders Analyses adjusted for age and total intracranial volumes. The results were corrected for multiple comparisons and considered statistically significant for an FDR-corrected *P* < 0.05 at the cluster level. Sagittal, axial and coronal views. FDR = false discovery rate; VBM = voxel-based morphometry

Two AUDs/NSDs were significantly associated with lower GM volumes (Table 3; Fig. 4): (i) dryness (nasal, ocular or oral) in the left insula; (ii) severe taste disorders in the left putamen and left caudate nucleus. Conversely, three AUDs/NSDs were significantly associated with greater GM volumes: (i) severe lacrimation; (ii) hypersalivation; and (iii) severe smell disorders (Table 3; Fig. 5). No other significant associations were found for the other AUDs/NSDs considered. Note that, in sensitivity analyses also adjusted for the visit corresponding to the last MRI, only the association between severe taste disorders and smaller GM volumes remained statistically significant in the putamen (left and right), in the caudate nucleus (left and right) and in the right accumbens nucleus.

**Table 3:** Whole-brain VBM analyses of grey matter volumes, comparing patients with or without autonomic or neurosensory disorders

	No. of symptomatic subjects	No. of clusters with qFDR < 0.05	Cluster size	qFDR	Peak MNI coordinates	Associated regions
AUDs/NSDs associated with decreased grey matter volume						
Nasal, ocular or oral dryness	89 (76.7%)	1	139	0.039	−40.5 −10.5 6	Insula (left)
Severe taste disorder	32 (27.6%)	1	182	0.006	−24 12 −7.5	Putamen, caudate nucleus (left)
AUDs/NSDs associated with increased grey matter volume						
Severe lacrimation	31 (26.7%)	4	201 165 165 139	0.012 0.012 0.012 0.021	19.5 −12 −30 −13.5 −48 39 28.5 −18 −16.5 15 −46.5 37.5	Parahippocampal gyrus, amygdala (right) Precuneus, middle cingulate and paracingulate gyri (left) Hippocampus, parahippocampal gyrus (right) Precuneus, middle cingulate and paracingulate gyri (right)
Hypersalivation	49 (42.2%)	3	437 421 152	0.000 0.000 0.017	33 −34.5 −4.5 34.5 −1.5 −22.5 −30 −25.5 −7.5	Hippocampus, parahippocampal gyrus (right) Amygdala, hippocampus, parahippocampal gyrus (right) Hippocampus, parahippocampal gyrus (left)
Severe smell disorder	37 (31.9%)	1	141	0.022	−33 −61.5 37.5	Inferior parietal gyrus, angular gyrus (left)

*AUDs* autonomic disorders, *FDR* false discovery rate, *MNI *Montreal Neurological Institute, *NSDs* neurosensory disorders, *VBM* voxel-based morphometry

## Discussion

### Interpretation in light of the literature

#### Screening for autonomic disorders

Although the existence of AUDs in DLB is widely recognized, research efforts seem very uneven depending on the type of disorder considered. While some symptoms, such as NOH, constipation and urinary dysfunction (i.e. those included in diagnostic recommendations) have been the subject of numerous studies, other AUDs seem to have been little studied:

- Orthostatic hypotension (OH) is one of the symptoms most frequently studied in DLB. Often subclinical [31], its detection requires objective measurements, which can also identify whether or not its origin is neurogenic. According to a meta-analysis of 18 studies published until 2022, the prevalence of OH (whether neurogenic or not) was estimated to be 51% among 662 DLB patients [32]. Nevertheless, given the fluctuating nature of OH, its prevalence may have been underestimated as most studies were based on a single screening. As an example, in our study sample, 25–27% of patients had NOH when a single screening time was considered while its prevalence increased to 69% when multiple screenings were considered. Repeated screening for OH in DLB seems essential given its value for the differential diagnosis with other neurocognitive diseases [33] and associated complications.
- Regarding constipation and urinary dysfunction, several studies were performed in DLB but reported highly variable prevalence rates, ranging from 28% to 83% for self-reported constipation [6–8, 11–13, 19, 34–39] and from 18% to 97% for self-reported urinary dysfunction [6, 10–13, 19, 34, 37, 39–41]. For the latter, this variability could be partly explained by the heterogeneity of the symptoms explored, including urinary urgency, urinary frequency, incomplete emptying, dysuria, nocturia and incontinence, etc. Conversely, the few data available on sexual dysfunction in DLB indicate prevalence rates ranging from 9% to 33% [7, 8, 13, 37] and also encompassing a wide variety of symptoms, including erectile dysfunction, ejaculation problems, change in libido, vaginal dryness, etc. In our study, a considerable proportion of patients reported constipation (67%), urinary dysfunction (68%) and sexual dysfunction (71%) and there appeared to be little fluctuation in the reports of these symptoms during follow-up. Future studies based on objective measurements (urodynamic tests, etc.) are needed to better characterize DLB symptoms and their origin (neurogenic or not), thus potentially increasing their usefulness for differential diagnosis purposes [6, 7, 11, 19, 36, 42] and for understanding the pathophysiology of the disease. Indeed, to our best knowledge, only one study has reported a tendency for an increased colonic transit time in DLB compared to PD [43], and only three studies based on urodynamic measurements in DLB patients have been published [42, 44, 45], which found that lower urinary tract symptoms were extremely common (> 90%).\
- Dryness or hypersecretion symptoms have been little studied in DLB. In the literature, the prevalence rates of dry mouth, hypersalivation and dry eyes seem to vary between 40% and 50% [6, 8, 19], 16% and 30% [7, 8, 11, 13] and 18% and 24% [6, 8], respectively, while our group also reported prevalence rates of dry nose (19%), rhinorrhoea (28%) and lacrimation (23%) in the MEMENTO cohort [8]. Interestingly, while patients’ reporting of these symptoms in our study seemed to fluctuate over the follow-up, all these symptoms were relatively frequent (all had a prevalence above 40% during the follow-up). Moreover, two symptoms stood out as the two most frequently reported: rhinorrhoea (79%) and dry mouth (73%). Interestingly, in PD, rhinorrhoea is suggested to be partially related to sympathetic denervation, and its prevalence was estimated to 45% in a recent meta-analysis (451 PD patients from six studies) [46], a result concordant with the prevalence of rhinorrhoea at 24 and 36 months in our study (49% and 41%, respectively). Regarding dry mouth, Beach et al. [47]. highlighted the presence of Lewy-type synucleinopathy in the submandibular glands of 71% of autopsied DLB patients, which may lead to reduced salivary secretion, as observed in PD [48]. Although rarely studied, these two symptoms could be good leads for improving differential diagnosis, as suggested by three studies for dry mouth [6, 8, 19] and one for rhinorrhoea [8].

#### Screening for neurosensory disorders

- Photophobia is a symptom commonly associated with ocular diseases, primary headaches and progressive supranuclear palsy [49, 50] (and has also been studied, albeit less frequently, in other neurodegenerative diseases, such as AD and PD) [51–53]. To our best knowledge, only one previous study assessed the presence of photophobia in DLB [8], reporting a prevalence of 36.3%, consistent with our findings at 24 and 36 months (38.4% and 39.4%, respectively). When the entire follow-up was taken into account, its prevalence increased to 59.9% including 32.4% with severe photophobia (i.e. permanent/with all types of light) and, in a previous article from our group in the same cohort [30], photophobia was associated with lower GM volumes in the right precentral cortex, in the eyelid motor region of Penfield’s homunculus. The frequency of photophobia in DLB subjects calls into question its usefulness for differential diagnosis with progressive supranuclear palsy (for which it is currently considered a supportive feature) [54] or the presence of tau co-pathology in patients in our study. Furthermore, although modest, the correlations found raise the question of a relationship between photophobia and eye dryness or lacrimation.
- Hyposmia is considered as a supportive clinical feature for the diagnosis of DLB, in part because various studies have highlighted the potential usefulness of olfactory testing for differential diagnosis with AD [10, 55]. In DLB, smell disorders are early-onset [11, 12, 56] and frequent. Previous studies based on questionnaires reported a prevalence of anosmia/hyposmia varying between 32% and 41% [9, 11, 12, 57], while Utsumi et al. [35] reported that over 68% of DLB patients presented with two smells were unable to either perceive or recognize them. Accordingly, in our study, 50% of patients reported a smell disorder at least once during follow-up and 34% a severe one. Surprisingly, reports of smell disorders seemed to fluctuate over the follow-up, raising the question of whether fluctuations were real or that patients were having difficulty in perceiving symptoms. In a study by Thomas et al. [9], 59% of subjects reporting no smell disorders had impaired performance in tests, while 21% of subjects reporting a smell disorder had preserved performances. If these results could suggest a difficulty in perceiving smell disorders, the presence of fluctuations could also have alternatively impacted the concordance between symptom reporting and objective measurements at a given time. As DLB is characterized by fluctuation in its core clinical features, clarifying whether this may also be the case for certain supportive clinical features seems to be a key factor in improving its diagnostic criteria.
- Unlike hyposmia, to our best knowledge, this is the first study to evaluate taste disorders in probable DLB patients, highlighting a prevalence of 43% over the follow-up. Prevalence rates at 24 and 36 months (17% in each case) were of the same order of magnitude as in PD, with prevalence rates varying between 20% and 30% [58]. Future studies based on objective measurement of taste capacity (as is the case in PD studies) will be needed to confirm these results, given the poor correlation between perception and objective measures of taste disorders reported in PD [59] and the potential impact of smell disorders on taste perception.

### Neuroanatomical correlates using VBM analysis

Investigating the neuroanatomical correlates of AUDs/NSDs in DLB, we highlighted associations between some symptoms and lower GM volumes using VBM techniques. First, dryness-type symptoms (either ocular, nasal or oral) were associated with lower GM volumes in the left insula, an area involved in the central control of autonomic functions [60–62]. Notably, the insula contains the primary interoceptive cortex receiving inputs from skin, visceral and gustatory receptors [60, 63]. Moreover, several studies suggest its implication in thirst management [64, 65] as well as in dry eye-related corneal neuropathic pain [66], representing potential links with dryness-type symptoms. Secondly, while no association was found with primary or secondary taste areas, severe taste disorders were associated with lower GM volumes in the putamen and caudate nucleus (as well as in the accumbens nucleus in our sensitivity analysis). Although not directly involved in taste perception [58], these structures are major players in reward processing, including taste-related rewards [67–69]. This could suggest that it is not the taste perception itself that is altered, but rather the “wanting” and “liking” of food [70, 71]. A detailed description of the taste disorders (decrease or increase in perception, changes in food pleasantness, etc.) reported by patients would be necessary to refine these results and better assess the plausibility of the association found. An alternative hypothesis could also be a co-occurrence of taste disorders and GM loss in the striatum in more advanced stages of the disease, with no causal relationship between these two phenomena. However, in our sample, subjects with taste disorders did not appear to be more severe compared to asymptomatic subjects (mean MMSE scores at the time of MRI = 24 and 23, respectively; p-value = 0.16).

More surprisingly, our VBM analyses also revealed greater GM volumes for certain symptoms. Firstly, severe lacrimation and hypersalivation were independently associated with greater GM volumes in the limbic system (including the hippocampus, the parahippocampal gyrus, the middle cingulate gyrus and the amygdala) and in the precuneus (only for severe lacrimation). Although phenomena such as lacrimation or hypersalivation have been reported in cases of temporal epilepsy [72, 73], these associations seem less obvious to explain and could be artefactual. Indeed, these associations were no longer significant when adjusting for the MRI timing. This could suggest a confounding given that participants who only had an MRI at inclusion were more likely to have hippocampal atrophy and no symptoms (as there was only one screening time point). Secondly, we highlighted an association between severe smell disorders and greater GM volumes in the left angular gyrus, a multisensory integration area [74, 75]. Interestingly, a previous study also reported a correlation between larger GM volumes in the angular gyrus and poorer olfactory test scores [76]. In this study, anosmic participants also presented an increased functional connectivity from the olfactory cortex to the angular gyrus and from the angular gyrus to area responsible for visual processing, suggesting a sensory compensatory mechanism. Nevertheless, this positive association should be interpreted with caution. Moreover, in our study, smell disorders were not associated with lower GM volumes in the olfactory bulbs or in other area involved in olfaction, despite the well-known presence of alpha-synuclein deposits in these structures [15, 77]. This could either reflect an olfactory dysfunction without GM volume loss, or a lack of sensitivity in our analysis (small sample size, absence of objective measures of olfaction, etc.).

Finally, we found no significant association between NOH or constipation and GM volumes, which seems consistent with studies suggesting that, in DLB, some AUDs are mainly associated with peripheral damage [33, 78]. Indeed, several anatomopathological studies [14, 16, 47, 78–81] have highlighted the presence of alpha-synuclein deposits in various tissues, including among others the spinal cord, the sympathetic and parasympathetic ganglionic neurons, the vagus nerve, the heart, the enteric nervous system and the gastro-intestinal tract, the pelvic plexus and the genitourinary tract, the retina, the submandibular gland and the adrenal gland. Nevertheless, our results cannot rule out the involvement of brain structures in these AUDs. Indeed, given the small size of our sample, the absence of objective measures for the AUDs studied, the limited sensitivity of standard parcellation approaches to brainstem nuclei and the potentially non-neurogenic origin of some of the symptoms reported, our study may have lacked the power to demonstrate atrophy of some central autonomic structures [78]. Moreover, at certain stages of the disease, alpha-synuclein deposits in the central nervous system may induce AUDs without co-existing neuronal loss, encouraging complementary approaches to the study of these symptoms (functional MRI, diffusion, etc.) [78]. 

### Strengths and limitations

To improve diagnostic accuracy, diagnoses of DLB were reassessed at the end of follow-up, taking advantage of the repeated clinical and neuropsychological assessments carried out by an experienced team. Nevertheless, although diagnoses were based on recommended clinical criteria, only a small proportion of diagnoses benefited from paraclinical examinations that would have helped reinforce their accuracy.

Regarding the collection of data on the presence or absence of AUDs/NSDs, our study benefited from standardized and prospective collection, thus limiting the risk of recall bias. Repeated screening for these symptoms (up to 11 visits) is one of the original features of our study, enabling us to illustrate their evolution over time. However, it also has certain limitations. Apart from testing for NOH, screening for AUDs/NSDs was based on participants’ self-reports, which may have varied according to their impact on quality of life, the patient’s cognitive state, etc. In particular, our results highlight a lower reporting of certain symptoms among subjects with dementia (compared to MCI) and among subjects taking cholinesterase inhibitors (who may have mixed dementia more frequently), suggesting a possible recall bias. Furthermore, a link between the cognitive fluctuations encountered in DLB and those highlighted in this article cannot be ruled out. Screening using objective measurements [18, 45, 58, 82] would have enabled a more accurate assessment and the detection of sub-clinical disorders. Finally, AUDs/NSDs are far from being specific to DLB, many other causes for these symptoms (medication, diabetes, other neurodegenerative diseases, etc.) exist. In particular, certain treatments that are common in our sample have adverse effects such as dry mouth or constipation (antidepressants) or lacrimation, rhinorrhoea, hypersalivation (cholinesterase inhibitors). Thus, the specific cause(s) of the symptoms cannot be easily identified.

An original feature of our study is to have investigated the neuroanatomical bases associated with specific AUDs/NSDs by means of VBM analyses. Nevertheless, given its modest sample size and the absence of objective measurements for most AUDs/NSDs, our study may have lacked the statistical power to highlight certain associations.

## Conclusion

In our sample of probable DLB patients at the MCI or dementia stages, 95.7% of patients reported at least one AUD and 76.8% at least one NSD. Among these disorders, while some symptoms have already been the subject of a number of studies and are part of the supportive clinical features for the diagnosis of DLB (i.e. hyposmia, constipation, urinary dysfunction and NOH), others remain little studied including some that turned out to be very frequent. Indeed, rhinorrhoea, dry mouth and sexual dysfunction were reported by 79.3%, 73.3% and 70.6% of our patients, respectively, and - should our results be confirmed by other studies - could represent interesting avenues to explore in future studies in order to assess their potential value in the diagnosis of DLB. Photophobia – a supportive clinical feature of progressive supranuclear palsy – was also found in 59.9% and taste disorders in 43.3% of patients. Furthermore, repeated screenings revealed a certain degree of fluctuation for some AUDs/NSDs. While only 25% of our sample had NOH when a single screening time was considered, its prevalence increased to 69% when the entire follow-up was considered, a reminder of the need for regular screening for NOH in DLB patients to prevent related complications. More surprisingly, self-reports of smell and taste disorders as well as dryness/hypersecretion symptoms also appeared to show some degree of fluctuation. Clarifying whether this is due to actual fluctuations (as is the case for DLB core clinical features) or to difficulty in symptom declaration seems essential for accurately assessing their potential added-value in the differential diagnosis. Finally, in searching for neuroanatomical correlates of AUDs/NSDs using VBM methods, we highlighted a potential involvement of the insula in dryness-type symptoms and of certain basal ganglia in severe taste disorders in DLB. To confirm and refine ours results, future studies combining self-reporting and objective measures, and attempting to better characterize the proportion of symptoms due to neurogenic damage seem essential and will help to better understand the potential diagnostic value of some AUDs/NSDs, their importance in terms of prevention, and their ability to shed light on the pathophysiology of DLB. The study of these symptoms using other neuroimaging techniques (functional MRI, diffusion, etc.) would also provide a better understanding of their origins.

## Supplementary Information

Supplementary Material 1

Supplementary Material 2

Supplementary Material 3

Supplementary Material 4

## Data Availability

The data that support the findings of this study are available from F. Blanc (f.blanc@unistra.fr), upon reasonable request.

## Abbreviations

AD: Alzheimer’s Disease
AUDs: Autonomic Disord ers
DLB: Dementia With Lewy Bodies
DTI: Diffusion Tensor Imaging
FDR: False Discovery Rate
GM: Grey Matter
IADL: Instrumental Activities of Daily Living
MCI: Mild Cognitive Impairment
MMSE: Mini-Mental State Examination
MNI: Montreal Neurological Institute
NOH: Neurogenic Orthostatic Hypotension
NSDs: Neurosensory Disorders
OH: Orthostatic Hypotension
PD: Parkinson’s Disease
Q1-Q3: 1 st and 3rd quartiles
RBD: Rapid Eye Movement Sleep Behaviour Disorder
SD: Standard Deviation
VBM: Voxel-based Morphometry

## References

[1] McKeith I, Mintzer J, Aarsland D, Burn D, Chiu H, Cohen-Mansfield J, et al. Dementia with Lewy bodies. Lancet Neurol. 2004;3(1):19–28. 14693108 10.1016/s1474-4422(03)00619-7

[2] Vann Jones SA, O’Brien JT. The prevalence and incidence of dementia with lewy bodies: a systematic review of population and clinical studies. Psychol Med. 2014;44(4):673–83. 23521899 10.1017/S0033291713000494

[3] Nelson PT, Jicha GA, Kryscio RJ, Abner EL, Schmitt FA, Cooper G, et al. Low sensitivity in clinical diagnoses of dementia with lewy bodies. J Neurol. 2010;257(3):359–66. 19795154 10.1007/s00415-009-5324-y PMC2839040

[4] McKeith IG, Boeve BF, Dickson DW, Halliday G, Taylor JP, Weintraub D, et al. Diagnosis and management of dementia with lewy bodies: fourth consensus report of the DLB consortium. Neurology. 2017;89(1):88–100. 28592453 10.1212/WNL.0000000000004058 PMC5496518

[5] McKeith IG, Ferman TJ, Thomas AJ, Blanc F, Boeve BF, Fujishiro H, et al. Research criteria for the diagnosis of prodromal dementia with lewy bodies. Neurology. 2020;94(17):743–55. 32241955 10.1212/WNL.0000000000009323 PMC7274845

[6] Hamilton CA, Frith J, Donaghy PC, Barker SAH, Durcan R, Lawley S, et al. Assessment of autonomic symptoms may assist with early identification of mild cognitive impairment with Lewy bodies. Int J Geriatr Psychiatry. 2022. 10.1002/gps.5703. 35302677 10.1002/gps.5703 PMC9311677

[7] Liu S, Liu C, Hu W, Ji Y. Frequency, severity, and duration of autonomic symptoms in patients of prodromal dementia with lewy bodies. J Alzheimers Dis. 2022;89(3):923–9. 35988221 10.3233/JAD-220275

[8] Blanc F, Bouteloup V, Paquet C, Chupin M, Pasquier F, Gabelle A, et al. Prodromal characteristics of dementia with Lewy bodies: baseline results of the MEMENTO memory clinics nationwide cohort. Alzheimers Res Ther. 2022;14(1):96. 35854388 10.1186/s13195-022-01037-0 PMC9295361

[9] Thomas AJ, Hamilton CA, Barker S, Durcan R, Lawley S, Barnett N, et al. Olfactory impairment in mild cognitive impairment with Lewy bodies and Alzheimer’s disease. Int Psychogeriatr. 2022;34(6):585–92. 34666863 10.1017/S1041610221001265

[10] Liu S, Jiang Z, Zhao J, Li Z, Li R, Qiu Y, et al. Disparity of smell tests in Alzheimer’s disease and other neurodegenerative disorders: a systematic review and meta-analysis. Front Aging Neurosci. 2023;15:1249512. 37744388 10.3389/fnagi.2023.1249512 PMC10512741

[11] Chiba Y, Fujishiro H, Iseki E, Ota K, Kasanuki K, Hirayasu Y, et al. Retrospective survey of prodromal symptoms in dementia with lewy bodies: comparison with Alzheimer’s disease. Dement Geriatr Cogn Disord. 2012;33(4):273–81. 22722638 10.1159/000339363

[12] Fujishiro H, Iseki E, Nakamura S, Kasanuki K, Chiba Y, Ota K, et al. <article-title update="added"> Dementia with <scp>L</scp> ewy bodies: early diagnostic challenges. Psychogeriatrics. 2013;13(2):128–38. 23909972 10.1111/psyg.12005

[13] Hu W, Liu S, Wang F, Zhu H, Du X, Ma L, et al. Autonomic symptoms are predictive of dementia with Lewy bodies. Parkinsonism Relat Disord. 2022;95:1–4. 34942564 10.1016/j.parkreldis.2021.11.023

[14] Wakabayashi K, Mori F, Tanji K, Orimo S, Takahashi H. Involvement of the peripheral nervous system in synucleinopathies, tauopathies and other neurodegenerative proteinopathies of the brain. Acta Neuropathol (Berl). 2010;120(1):1–12. 20532896 10.1007/s00401-010-0706-x

[15] Cersosimo MG. Propagation of alpha-synuclein pathology from the olfactory bulb: possible role in the pathogenesis of dementia with lewy bodies. Cell Tissue Res. 2018;373(1):233–43. 29196808 10.1007/s00441-017-2733-6

[16] Gelpi E, Navarro-Otano J, Tolosa E, Gaig C, Compta Y, Rey MJ, et al. <article-title update="added">Multiple organ involvement by alpha‐synuclein pathology in Lewy body disorders. Mov Disord. 2014;29(8):1010–8. 24395122 10.1002/mds.25776

[17] Borghammer P, Horsager J, Andersen K, Van Den Berge N, Raunio A, Murayama S, et al. Neuropathological evidence of body-first vs. brain-first Lewy body disease. Neurobiol Dis. 2021;161:105557. 34763110 10.1016/j.nbd.2021.105557

[18] Clement G, Cavillon G, Vuillier F, Bouhaddi M, Béreau M. Unveiling autonomic failure in synucleinopathies: significance in diagnosis and treatment. Rev Neurol (Paris). 2024;180(1–2):79–93. 38216420 10.1016/j.neurol.2023.12.004

[19] Allan L, McKeith I, Ballard C, Kenny RA. The prevalence of autonomic symptoms in dementia and their association with physical activity, activities of daily living and quality of life. Dement Geriatr Cogn Disord. 2006;22(3):230–7. 16902277 10.1159/000094971

[20] Del Pino R, Murueta-Goyena A, Acera M, Carmona-Abellan M, Tijero B, Lucas-Jiménez O, et al. Autonomic dysfunction is associated with neuropsychological impairment in lewy body disease. J Neurol. 2020;267(7):1941–51. 32170444 10.1007/s00415-020-09783-7

[21] Pilotto A, Romagnolo A, Tuazon JA, Vizcarra JA, Marsili L, Zibetti M, et al. Orthostatic hypotension and REM sleep behaviour disorder: impact on clinical outcomes in α-synucleinopathies. J Neurol Neurosurg Psychiatry. 2019;90(11):1257–63. 31142660 10.1136/jnnp-2019-320846

[22] Mendoza-Velásquez JJ, Flores-Vázquez JF, Barrón-Velázquez E, Sosa-Ortiz AL, Illigens BMW, Siepmann T. Autonomic dysfunction in α-Synucleinopathies. Front Neurol. 2019;10:363. 31031694 10.3389/fneur.2019.00363 PMC6474181

[23] Bousiges O, Philippi N, Lavaux T, Perret-Liaudet A, Lachmann I, Schaeffer-Agalède C, et al. Differential diagnostic value of total alpha-synuclein assay in the cerebrospinal fluid between Alzheimer’s disease and dementia with Lewy bodies from the prodromal stage. Alzheimers Res Ther. 2020;12(1):120. 32993772 10.1186/s13195-020-00684-5 PMC7523311

[24] Bousiges O, Cretin B, Lavaux T, Philippi N, Jung B, Hezard S, et al. Diagnostic value of cerebrospinal fluid biomarkers (Phospho-Tau181, total-Tau, Aβ42, and Aβ40) in prodromal stage of Alzheimer’s disease and dementia with Lewy bodies. J Alzheimers Dis. 2016;51(4):1069–83. 26923009 10.3233/JAD-150731

[25] Dubois B, Feldman HH, Jacova C, Dekosky ST, Barberger-Gateau P, Cummings J, et al. Research criteria for the diagnosis of Alzheimer’s disease: revising the NINCDS-ADRDA criteria. Lancet Neurol. 2007;6(8):734–46. 17616482 10.1016/S1474-4422(07)70178-3

[26] Ferman TJ, Boeve BF, Smith GE, Lin SC, Silber MH, Pedraza O, et al. Inclusion of RBD improves the diagnostic classification of dementia with lewy bodies. Neurology. 2011;77(9):875–82. 21849645 10.1212/WNL.0b013e31822c9148 PMC3162640

[27] Fénelon G, Soulas T, Zenasni F, de Cleret Langavant L. The changing face of Parkinson’s disease-associated psychosis: a cross-sectional study based on the new NINDS-NIMH criteria. Mov Disord Off J Mov Disord Soc. 2010;25(6):763–6. 10.1002/mds.22839 PMC2891710 20437542

[28] Gjerstad MD, Boeve B, Wentzel-Larsen T, Aarsland D, Larsen JP. Occurrence and clinical correlates of REM sleep behaviour disorder in patients with parkinson’s disease over time. J Neurol Neurosurg Psychiatry. 2008;79(4):387–91. 17557796 10.1136/jnnp.2007.116830

[29] Good CD, Johnsrude IS, Ashburner J, Henson RN, Friston KJ, Frackowiak RS. A voxel-based morphometric study of ageing in 465 normal adult human brains. Neuroimage. 2001;14(1 Pt 1):21–36. 11525331 10.1006/nimg.2001.0786

[30] Tisserand A, Cretin B, Mondino M, Botzung A, Sanna L, Demuynck C, et al. Neural correlates of photophobia in prodromal and mild dementia with Lewy bodies. Eur J Neurol. 2023;30(8):2215–21. 37154398 10.1111/ene.15829

[31] Bengtsson-Lindberg M, Larsson V, Minthon L, Wattmo C, Londos E. Lack of orthostatic symptoms in dementia patients with orthostatic hypotension. Clin Auton Res Off J Clin Auton Res Soc. 2015;25(2):87–94. 10.1007/s10286-014-0244-z 24743866

[32] Isik AT, Dost FS, Yavuz I, Ontan MS, Ates Bulut E, Kaya D. Orthostatic hypotension in dementia with lewy bodies: a meta-analysis of prospective studies. Clin Auton Res Off J Clin Auton Res Soc. 2023;33(2):133–41. 10.1007/s10286-023-00933-1 36862320

[33] Leys F, Wenning GK, Fanciulli A. The role of cardiovascular autonomic failure in the differential diagnosis of α-synucleinopathies. Neurol Sci Off J Ital Neurol Soc Ital Soc Clin Neurophysiol. 2022;43(1):187–98. 10.1007/s10072-021-05746-6 PMC8724069 34817726

[34] Horimoto Y, Matsumoto M, Akatsu H, Ikari H, Kojima K, Yamamoto T, et al. Autonomic dysfunctions in dementia with Lewy bodies. J Neurol. 2003;250(5):530–3. 12736730 10.1007/s00415-003-1029-9

[35] Utsumi K, Fukatsu R, Yamada R, Takamaru Y, Hara Y, Yasumura S. Characteristics of initial symptoms and symptoms at diagnosis in probable dementia with lewy body disease: incidence of symptoms and gender differences. Psychogeriatrics. 2020;20(5):737–45. 32743894 10.1111/psyg.12586

[36] Camerucci E, Mullan AF, Bower JH, Bharucha AE, Turcano P, Stang CD, et al. Lifelong constipation in Parkinson’s disease and other clinically defined alpha-synucleinopathies: a population-based study in Southeast Minnesota. Parkinsonism Relat Disord. 2023;107:105244. 36630736 10.1016/j.parkreldis.2022.105244 PMC10262204

[37] Bae HJ, Cheon SM, Kim JW. Autonomic dysfunctions in parkinsonian disorders. J Mov Disord. 2009;2(2):72–7. 24868361 10.14802/jmd.09019 PMC4027718

[38] Stubendorff K, Aarsland D, Minthon L, Londos E. The impact of autonomic dysfunction on survival in patients with dementia with Lewy bodies and Parkinson’s disease with dementia. PLoS One. 2012;7(10):e45451. 23049679 10.1371/journal.pone.0045451 PMC3462171

[39] Soysal P, Koc Okudur S, Uslu F, Smith L. Functional loss and worsening geriatric assessment parameters are more common in dementia with lewy bodies than Alzheimer’s disease. Psychogeriatrics. 2023;23(1):77–85. 36349708 10.1111/psyg.12905

[40] Gan J, Chen Z, Liu S, Shi Z, Liu Y, Wang XD, et al. The presence and co-incidence of geriatric syndromes in older patients with mild-moderate lewy body dementia. BMC Neurol. 2022;22(1):355. 36123648 10.1186/s12883-022-02897-7 PMC9484208

[41] Naharci MI, Kayahan Satis N, Ozsurekci C, Tasci I. Assessment of clinical features and coexisting geriatric syndromes in newly diagnosed dementia with lewy bodies: a retrospective study in a tertiary geriatrics setting in Turkey. Eur Geriatr Med. 2023;14(1):19–27. 36512254 10.1007/s41999-022-00727-0

[42] Ransmayr GN, Holliger S, Schletterer K, Heidler H, Deibl M, Poewe W, et al. Lower urinary tract symptoms in dementia with Lewy bodies, Parkinson disease, and Alzheimer disease. Neurology. 2008;70(4):299–303. 18209204 10.1212/01.wnl.0000296826.61499.26

[43] Doi H, Sakakibara R, Masuda M, Tateno F, Aiba Y, Kishi M, et al. Gastrointestinal function in dementia with Lewy bodies: a comparison with Parkinson disease. Clin Auton Res. 2019. 10.1007/s10286-019-00597-w. 30741396 10.1007/s10286-019-00597-w

[44] Sakakibara R, Ito T, Uchiyama T, Asahina M, Liu Z, Yamamoto T, et al. Lower urinary tract function in dementia of lewy body type. J Neurol Neurosurg Psychiatry. 2005;76(5):729–32. 15834036 10.1136/jnnp.2004.046243 PMC1739636

[45] Tateno F, Sakakibara R, Ogata T, Kishi M, Tsuyusaki Y, Takahashi O, et al. Lower urinary tract function in dementia with Lewy bodies (DLB). Mov Disord. 2015;30(3):411–5. 25356960 10.1002/mds.25985

[46] Chen T, Edwards TS, Hinson VK, Soler ZM. Prevalence of rhinorrhea in Parkinson disease. Neurol Clin Pract. 2022;12(4):e75–81. 36382119 10.1212/CPJ.0000000000001180 PMC9647823

[47] Beach TG, Adler CH, Serrano G, Sue LI, Walker DG, Dugger BN, et al. Prevalence of Submandibular Gland Synucleinopathy in Parkinson’s Disease, Dementia with Lewy Bodies and other Lewy Body Disorders. J Parkinsons Dis. 2016;6(1):153–63. 26756744 10.3233/JPD-150680 PMC5498170

[48] Del Tredici K, Hawkes CH, Ghebremedhin E, Braak H. Lewy pathology in the submandibular gland of individuals with incidental Lewy body disease and sporadic Parkinson’s disease. Acta Neuropathol. 2010;119(6):703–13. 20229352 10.1007/s00401-010-0665-2

[49] Baschieri F, Vitiello M, Cortelli P, Calandra-Buonaura G, Morgante F. Autonomic dysfunction in progressive supranuclear palsy. J Neurol. 2023;270(1):109–29. 36042018 10.1007/s00415-022-11347-w PMC9813233

[50] Wu Y, Hallett M. Photophobia in neurologic disorders. Transl Neurodegener. 2017;6(1):26. 28932391 10.1186/s40035-017-0095-3 PMC5606068

[51] Bargagli A, Fontanelli E, Zanca D, Castelli I, Rosini F, Maddii S, et al. Neurophthalmologic and orthoptic ambulatory assessments reveal ocular and visual changes in patients with early alzheimer and parkinson’s disease. Front Neurol. 2020;11:577362. 33224092 10.3389/fneur.2020.577362 PMC7669827

[52] Barrell K, Bureau B, Turcano P, Phillips GD, Anderson JS, Malik A, et al. High-Order visual Processing, visual Symptoms, and visual hallucinations: A possible symptomatic progression of parkinson’s disease. Front Neurol. 2018;9:999. 30538666 10.3389/fneur.2018.00999 PMC6277574

[53] Mohanty D, Hay KR, Berkowitz S, Patel S, Lin YC, Kang H, et al. Clinical implications of photophobia in progressive supranuclear palsy. Clin Park Relat Disord. 2021;4:100097. 34316674 10.1016/j.prdoa.2021.100097 PMC8299976

[54] Hoglinger GU, Respondek G, Stamelou M, Kurz C, Josephs KA, Lang AE, et al. Clinical diagnosis of progressive supranuclear palsy: the movement disorder society criteria. Mov Disord Off J Mov Disord Soc. 2017;32(6):853–64. 10.1002/mds.26987 PMC5516529 28467028

[55] Beach TG, Adler CH, Zhang N, Serrano GE, Sue LI, Driver-Dunckley E, et al. Severe hyposmia distinguishes neuropathologically confirmed dementia with Lewy bodies from Alzheimer’s disease dementia. PLoS One. 2020;15(4):e0231720. 32320406 10.1371/journal.pone.0231720 PMC7176090

[56] Fereshtehnejad SM, Yao C, Pelletier A, Montplaisir JY, Gagnon JF, Postuma RB. Evolution of prodromal Parkinson’s disease and dementia with Lewy bodies: a prospective study. Brain J Neurol. 2019;142(7):2051–67. 10.1093/brain/awz111 31111143

[57] McShane RH, Nagy Z, Esiri MM, King E, Joachim C, Sullivan N, et al. Anosmia in dementia is associated with lewy bodies rather than alzheimer’s pathology. J Neurol Neurosurg Psychiatry. 2001;70(6):739–43. 11385006 10.1136/jnnp.70.6.739 PMC1737382

[58] Cecchini MP, Fasano A, Boschi F, Osculati F, Tinazzi M. Taste in Parkinson’s disease. J Neurol. 2015;262(4):806–13. 25280862 10.1007/s00415-014-7518-1

[59] Cecchini MP, Osculati F, Ottaviani S, Boschi F, Fasano A, Tinazzi M. Taste performance in Parkinson’s disease. J Neural Transm. 2014;121(2):119–22. 24078166 10.1007/s00702-013-1089-7

[60] Cersosimo MG, Benarroch EE. Central control of autonomic function and involvement in neurodegenerative disorders, vol. 117. 2013. p. 45–57. 10.1016/B978-0-444-53491-0.00005-5 24095115

[61] Conti M, Garasto E, Bovenzi R, Ferrari V, Mercuri NB, Di Giuliano F, et al. Insular and limbic abnormal functional connectivity in early-stage Parkinson’s disease patients with autonomic dysfunction. Cereb Cortex. 2024;34(7):bhae270. 38967041 10.1093/cercor/bhae270 PMC11909796

[62] Li G, Chen Z, Zhou L, Zhao A, Niu M, Li Y, et al. Altered structure and functional connectivity of the central autonomic network in idiopathic rapid eye movement sleep behaviour disorder. J Sleep Res. 2021;30(3):e13136. 32608031 10.1111/jsr.13136

[63] Sörös P, Inamoto Y, Martin RE. Functional brain imaging of swallowing: an activation likelihood estimation meta-analysis. Hum Brain Mapp. 2008;30(8):2426–39. 10.1002/hbm.20680 PMC6871071 19107749

[64] Saker P, Farrell MJ, Adib FRM, Egan GF, McKinley MJ, Denton DA. Regional brain responses associated with drinking water during thirst and after its satiation. Proc Natl Acad Sci U S A. 2014;111(14):5379–84. 24706817 10.1073/pnas.1403382111 PMC3986125

[65] Denton D, Shade R, Zamarippa F, Egan G, Blair-West J, McKinley M, et al. Neuroimaging of genesis and satiation of thirst and an interoceptor-driven theory of origins of primary consciousness. Proc Natl Acad Sci U S A. 1999;96(9):5304–9. 10220461 10.1073/pnas.96.9.5304 PMC21859

[66] Xu R, Zhang Y, Gu Q, Yuan T, Fan B, Xia J, et al. Alteration of neural activity and neuroinflammatory factors in the insular cortex of mice with corneal neuropathic pain. Genes Brain Behav. 2023;22(2):e12842. 36889983 10.1111/gbb.12842 PMC10067426

[67] Skałbania J, Tanajewski Ł, Furtak M, Hare TA, Wypych M. Pre-choice midbrain fluctuations affect self-control in food choice: a functional magnetic resonance imaging (fMRI) study. Cogn Affect Behav Neurosci. 2024. 10.3758/s13415-024-01231-7. 39379768 10.3758/s13415-024-01231-7 PMC11906498

[68] Norgren R, Hajnal A, Mungarndee SS. Gustatory reward and the nucleus accumbens. Physiol Behav. 2006;89(4):531–5. 16822531 10.1016/j.physbeh.2006.05.024 PMC3114426

[69] Yamamoto T. Neural substrates for the processing of cognitive and affective aspects of taste in the brain. Arch Histol Cytol. 2006;69(4):243–55. 17287579 10.1679/aohc.69.243

[70] Morales I, Berridge KC. ‘Liking’ and ‘wanting’ in eating and food reward: brain mechanisms and clinical implications. Physiol Behav. 2020;227:113152. 32846152 10.1016/j.physbeh.2020.113152 PMC7655589

[71] Frank GKW, Oberndorfer TA, Simmons AN, Paulus MP, Fudge JL, Yang TT, et al. Sucrose activates human taste pathways differently from artificial sweetener. Neuroimage. 2008;39(4):1559–69. 18096409 10.1016/j.neuroimage.2007.10.061

[72] Blumberg J, Fernández IS, Vendrame M, Oehl B, Tatum WO, Schuele S, et al. Dacrystic seizures: demographic, semiologic, and etiologic insights from a multicenter study in long-term video-EEG monitoring units. Epilepsia. 2012;53(10):1810–9. 22780551 10.1111/j.1528-1167.2012.03578.x PMC6294572

[73] Shah J, Zhai H, Fuerst D, Watson C. Hypersalivation in temporal lobe epilepsy. Epilepsia. 2006;47(3):644–51. 16529634 10.1111/j.1528-1167.2006.00480.x

[74] Joshi A, Hornstein H, Thaploo D, Faria V, Warr J, Hummel T. Neural processing of odors with different well-being associations—findings from two consecutive neuroimaging studies. Brain Sci. 2023;13(4):576. 37190541 10.3390/brainsci13040576 PMC10136803

[75] Li Z, Li Sbin, Tan S, Liu Llu, Yan C, Zou Lquan. Neural correlates of olfactory working memory in the human brain. Neuroimage. 2025;306:121005. 39788337 10.1016/j.neuroimage.2025.121005

[76] Iravani B, Peter MG, Arshamian A, Olsson MJ, Hummel T, Kitzler HH, et al. Acquired olfactory loss alters functional connectivity and morphology. Sci Rep. 2021;11:16422. 34385571 10.1038/s41598-021-95968-7 PMC8361122

[77] Beach TG, White CL, Hladik CL, Sabbagh MN, Connor DJ, Shill HA, et al. Olfactory bulb alpha-synucleinopathy has high specificity and sensitivity for lewy body disorders. Acta Neuropathol (Berl). 2009;117(2):169–74. 18982334 10.1007/s00401-008-0450-7 PMC2631085

[78] Coon EA. Autonomic dysfunction in the synucleinopathies. Semin Neurol. 2020;40(5):492–501. 32906169 10.1055/s-0040-1713844

[79] Nardone R, Höller Y, Brigo F, Versace V, Sebastianelli L, Florea C, et al. Spinal cord involvement in Lewy body-related α-synucleinopathies. J Spinal Cord Med. 2020;43(6):832–45. 30620687 10.1080/10790268.2018.1557863 PMC7808259

[80] Beach TG, Adler CH, Sue LI, Vedders L, Lue LF, White CL III, et al. Multi-organ distribution of phosphorylated alpha-synuclein histopathology in subjects with lewy body disorders. Acta Neuropathol (Berl). 2010;119(6):689–702. 20306269 10.1007/s00401-010-0664-3 PMC2866090

[81] de Hart Ruyter FJ, Evers MJAP, Morrema THJ, Dijkstra AA, den Haan J, Twisk JWR, et al. Neuropathological hallmarks in the post-mortem retina of neurodegenerative diseases. Acta Neuropathol. 2024;148(1):24. 39160362 10.1007/s00401-024-02769-z PMC11333524

[82] Metta V, Chung-Faye G, Ts Benamer H, Mrudula R, Goyal V, Falup-Pecurariu C, et al. Hiccups, hypersalivation, hallucinations in Parkinson’s disease: new insights, mechanisms, pathophysiology, and management. J Pers Med. 2023;13(5):711. 37240881 10.3390/jpm13050711 PMC10219524

