# Supplementary material for: Autonomic and neurosensory disorders in dementia with lewy bodies: prevalence and neural basis in the AlphaLewyMA cohort
Source: Alzheimers Res Ther. 2025 Dec 19;17:271. doi: 10.1186/s13195-025-01935-z (PMC12751402; doi:10.1186/s13195-025-01935-z)
Supplement: Supplementary file 1 — Supplementary Material 1 [file 13195_2025_1935_MOESM1_ESM.docx]

## Additional file 1: Definitions used for DLB diagnoses

| Parkinsonism | Using the Unified Parkinson’s Disease Rating Scale Part III, parkinsonism was defined as the presence of at least one of the following criteria:  - Rigidity  - Akinesia  - Resting or attitude tremor |
| --- | --- |
| Fluctuations | Using the Mayo Clinic Fluctuations Scale, participants were considered to have fluctuations if they or their companion (on the participant’s behalf) answered yes to at least two of the following questions:  - Are you sleepy or lethargic during the day, when you got enough sleep the night before?  - Do you sleep more than 2 hours during the day (before 7 p.m.)?  - Do you ever stare for long periods of time?  - Do you have episodes during the day when the flow of your ideas is disorganized, unclear or illogical? |
| Visual hallucinations | Using the Parkinson’s disease-associated psychotic symptoms questionnaire, participants were considered to have visual hallucinations if they or their companion (on the participant’s behalf) answered yes to the following question:  - Have you ever seen people, animals, objects, shadows or lights, etc., things that didn’t exist, that others couldn’t see, that weren’t really there? |
| Rapid eye movement sleep behaviour disorder (RBD) | Using a questionnaire adapted from Gjerstad et al., participants were considered to have RBD if they or their companion (on the participant’s behalf) answered yes to both the following questions:   - Do you move while you sleep? - Do you have frequent vivid dreams and nightmares? |
