# Supplementary material for: Autonomic and neurosensory disorders in dementia with lewy bodies: prevalence and neural basis in the AlphaLewyMA cohort
Source: Alzheimers Res Ther. 2025 Dec 19;17:271. doi: 10.1186/s13195-025-01935-z (PMC12751402; doi:10.1186/s13195-025-01935-z)
Supplement: Supplementary file 2 — Supplementary Material 2 [file 13195_2025_1935_MOESM2_ESM.docx]

**Additional file 2: Flow chart**

**
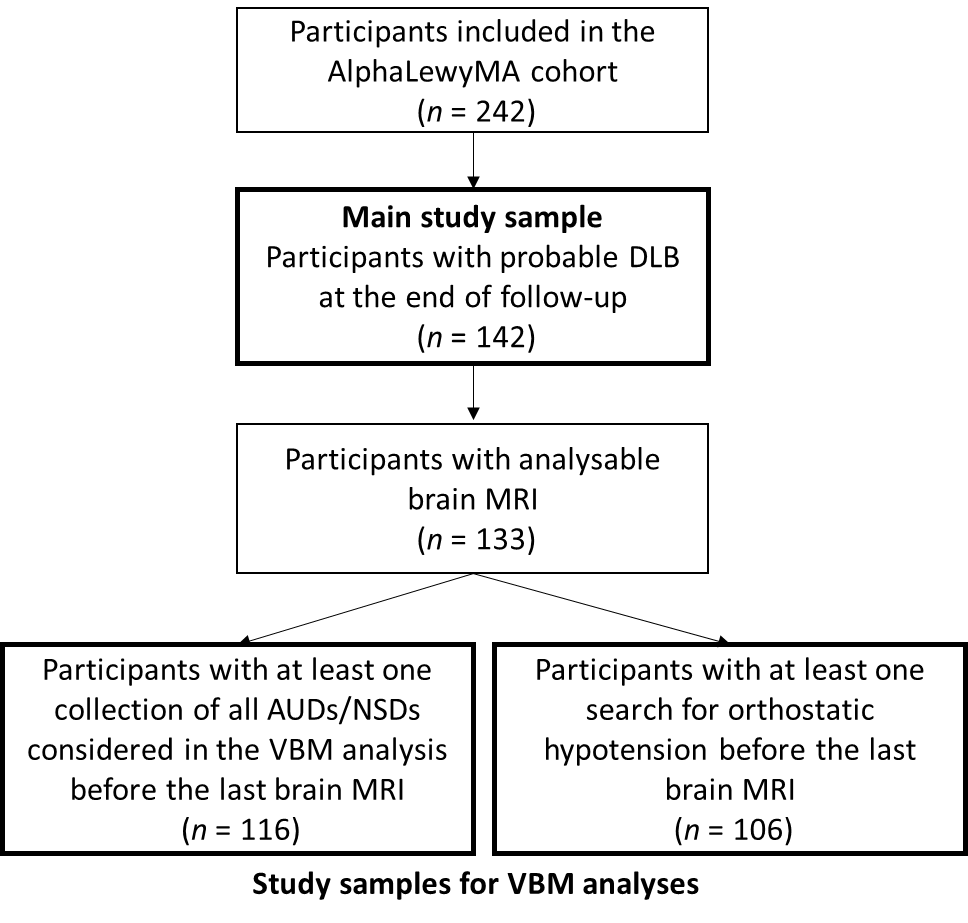
**

Figure: Flow chart. AlphaLewyMA cohort, France

AUDs = autonomic disorders; DLB = dementia with Lewy bodies; MRI = magnetic resonance imaging; NSDs = neurosensory disorders; VBM = voxel-based morphometry.
