# Supplementary material for: Autonomic and neurosensory disorders in dementia with lewy bodies: prevalence and neural basis in the AlphaLewyMA cohort
Source: Alzheimers Res Ther. 2025 Dec 19;17:271. doi: 10.1186/s13195-025-01935-z (PMC12751402; doi:10.1186/s13195-025-01935-z)
Supplement: Supplementary file 3 — Supplementary Material 3 [file 13195_2025_1935_MOESM3_ESM.docx]

## **Additional file 3:**

1. **Prevalence of autonomic and neurosensory disorders at 36 months stratified on clinical stage**


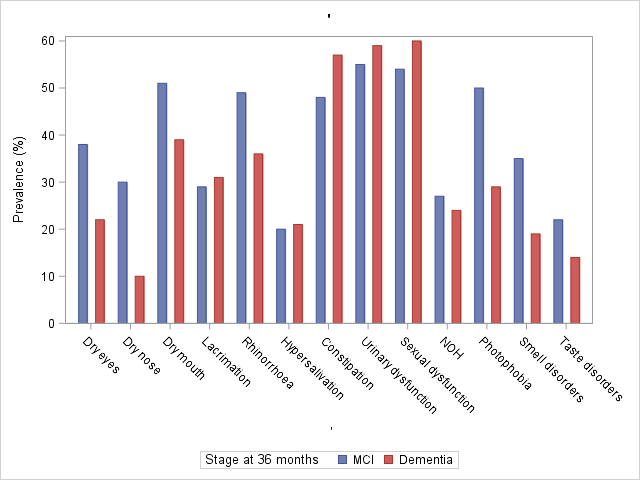


|  | | **Total (N=83)** | **MCI (N=41)** | **Dementia (N=42)** | **p-value** |
| --- | --- | --- | --- | --- | --- |
| Dry eyes | Missing data | 5 (6.0%) | 4 (9.8%) | 1 (2.4%) | p = 0.12 |
|  | No | 55 (70.5%) | 23 (62.2%) | 32 (78.0%) |  |
|  | Yes | 23 (29.5%) | 14 (37.8%) | 9 (22.0%) |  |
| Dry nose | Missing data | 5 (6.0%) | 4 (9.8%) | 1 (2.4%) | **p = 0.03** |
|  | No | 63 (80.8%) | 26 (70.3%) | 37 (90.2%) |  |
|  | Yes | 15 (19.2%) | 11 (29.7%) | 4 (9.8%) |  |
| Dry mouth | Missing data | 5 (6.0%) | 4 (9.8%) | 1 (2.4%) | p = 0.27 |
|  | No | 43 (55.1%) | 18 (48.6%) | 25 (61.0%) |  |
|  | Yes | 35 (44.9%) | 19 (51.4%) | 16 (39.0%) |  |
| Lacrimation | Missing data | 0 (0.0%) | 0 (0.0%) | 0 (0.0%) | p = 0.87 |
|  | No | 58 (69.9%) | 29 (70.7%) | 29 (69.0%) |  |
|  | Yes | 25 (30.1%) | 12 (29.3%) | 13 (31.0%) |  |
| Rhinorrhoea | Missing data | 0 (0.0%) | 0 (0.0%) | 0 (0.0%) | p = 0.23 |
|  | No | 48 (57.8%) | 21 (51.2%) | 27 (64.3%) |  |
|  | Yes | 35 (42.2%) | 20 (48.8%) | 15 (35.7%) |  |
| Hypersalivation | Missing data | 0 (0.0%) | 0 (0.0%) | 0 (0.0%) | p = 0.82 |
|  | No | 66 (79.5%) | 33 (80.5%) | 33 (78.6%) |  |
|  | Yes | 17 (20.5%) | 8 (19.5%) | 9 (21.4%) |  |
| Constipation | Missing data | 1 (1.2%) | 1 (2.4%) | 0 (0.0%) | p = 0.38 |
|  | No | 39 (47.6%) | 21 (52.5%) | 18 (42.9%) |  |
|  | Yes | 43 (52.4%) | 19 (47.5%) | 24 (57.1%) |  |
| Sexual dysfunction | Missing data | 23 (27.7%) | 6 (14.6%) | 17 (40.5%) | p = 0.66 |
|  | No | 26 (43.3%) | 16 (45.7%) | 10 (40.0%) |  |
|  | Yes | 34 (56.7%) | 19 (54.3%) | 15 (60.0%) |  |
| Urinary dysfunction | Missing data | 18 (21.7%) | 8 (19.5%) | 10 (23.8%) | p = 0.69 |
|  | No | 28 (43.1%) | 15 (45.5%) | 13 (40.6%) |  |
|  | Yes | 37 (56.9%) | 18 (54.5%) | 19 (59.4%) |  |
| NOH | Missing data | 20 (24.1%) | 11 (26.8%) | 9 (21.4%) | p = 0.83 |
|  | No | 47 (74.6%) | 22 (73.3%) | 25 (75.8%) |  |
|  | Yes | 16 (25.4%) | 8 (26.7%) | 8 (24.2%) |  |
| Photophobia | Missing data | 1 (1.2%) | 1 (2.4%) | 0 (0.0%) | **p = 0.046** |
|  | No | 50 (61.0%) | 20 (50.0%) | 30 (71.4%) |  |
|  | Yes | 32 (39.0%) | 20 (50.0%) | 12 (28.6%) |  |
|  |  |  |  |  |  |
| Smell disorders | Missing data | 4 (4.8%) | 4 (9.8%) | 0 (0.0%) | p = 0.11 |
|  | No | 58 (73.4%) | 24 (64.9%) | 34 (81.0%) |  |
|  | Yes | 21 (26.6%) | 13 (35.1%) | 8 (19.0%) |  |
| Taste disorders | Missing data | 4 (4.8%) | 4 (9.8%) | 0 (0.0%) | p = 0.39 |
|  | No | 65 (82.3%) | 29 (78.4%) | 36 (85.7%) |  |
|  | Yes | 14 (17.7%) | 8 (21.6%) | 6 (14.3%) |  |

1. **Prevalence of autonomic and neurosensory disorders at 24 months in 15 healthy controls included in the AlphaLewyMA cohort**

|  | **Prevalence at 24 months (n=15)** | |
| --- | --- | --- |
|  | **Missing** | **Present** |
| **Dry eyes** | 0 (0.0%) | **2 (13.3%)** |
| **Dry nose** | 0 (0.0%) | 0 (0.0%) |
| **Dry mouth** | 0 (0.0%) | 1 (6.7%) |
| **Lacrimation** | 0 (0.0%) | 0 (0.0%) |
| *including severe grade* |  | 0 (0.0%) |
| **Rhinorrhoea** | 0 (0.0%) | 1 (6.7%) |
| *including severe grade* |  | 1 (6.7%) |
| **Hypersalivation** | 0 (0.0%) | 0 (0.0%) |
| *including severe grade* |  | 0 (0.0%) |
| **Constipation** | 0 (0.0%) | 0 (0.0%) |
| *including severe grade* |  | 0 (0.0%) |
| **Sexual dysfunction** | 1 (6.7%) | **2 (14.3%)** |
| *including severe grade* |  | **2 (14.3%)** |
| **Neurogenic orthostatic hypotension** | 0 (0.0%) | **3 (20.0%)** |
| *including severe grade* |  | 0 (0.0%) |
| **Photophobia** | 0 (0.0%) | 1 (6.7%) |
| *including severe grade* |  | 1 (6.7%) |
| **Smell disorders** | 0 (0.0%) | 0 (0.0%) |
| *including severe grade* |  | 0 (0.0%) |
| **Taste disorders** | 0 (0.0%) | 1 (6.7%) |
| *including severe grade* |  | 1 (6.7%) |

1. **Mean number of autonomic and neurosensory disorders at each follow-up**


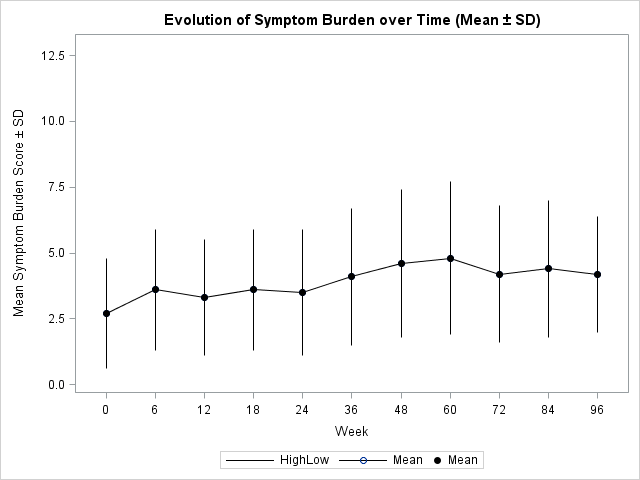


1. **Prevalence of autonomic and neurosensory disorders during the follow-up stratified by the intake of antidepressants**


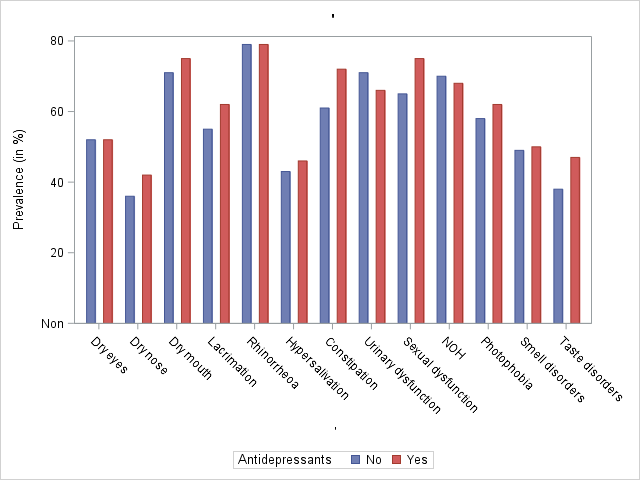


|  | | **Total (N=142)** | **No antidepressants (N=64)** | **Antidepressants (N=78)** | **p-value** |
| --- | --- | --- | --- | --- | --- |
| Dry eyes | Missing data | 11 (7.7%) | 6 (9.4%) | 5 (6.4%) | p = 0.97 |
|  | No | 63 (48.1%) | 28 (48.3%) | 35 (47.9%) |  |
|  | Yes | 68 (51.9%) | 30 (51.7%) | 38 (52.1%) |  |
| Dry nose | Missing data | 11 (7.7%) | 6 (9.4%) | 5 (6.4%) | p = 0.47 |
|  | No | 79 (60.3%) | 37 (63.8%) | 42 (57.5%) |  |
|  | Yes | 52 (39.7%) | 21 (36.2%) | 31 (42.5%) |  |
| Dry mouth | Missing data | 11 (7.7%) | 6 (9.4%) | 5 (6.4%) | p = 0.55 |
|  | No | 35 (26.7%) | 17 (29.3%) | 18 (24.7%) |  |
|  | Yes | 96 (73.3%) | 41 (70.7%) | 55 (75.3%) |  |
| Lacrimation | Missing data | 10 (7.0%) | 6 (9.4%) | 4 (5.1%) | p = 0.42 |
|  | No | 54 (40.9%) | 26 (44.8%) | 28 (37.8%) |  |
|  | Yes | 78 (59.1%) | 32 (55.2%) | 46 (62.2%) |  |
| Rhinorrhoea | Missing data | 2 (1.4%) | 2 (3.1%) | 0 (0.0%) | p = 0.95 |
|  | No | 29 (20.7%) | 13 (21.0%) | 16 (20.5%) |  |
|  | Yes | 111 (79.3%) | 49 (79.0%) | 62 (79.5%) |  |
| Hypersalivation | Missing data | 6 (4.2%) | 4 (6.3%) | 2 (2.6%) | p = 0.75 |
|  | No | 75 (55.1%) | 34 (56.7%) | 41 (53.9%) |  |
|  | Yes | 61 (44.9%) | 26 (43.3%) | 35 (46.1%) |  |
| Constipation | Missing data | 5 (3.5%) | 3 (4.7%) | 2 (2.6%) | p = 0.15 |
|  | No | 45 (32.8%) | 24 (39.3%) | 21 (27.6%) |  |
|  | Yes | 92 (67.2%) | 37 (60.7%) | 55 (72.4%) |  |
| Sexual dysfunction | Missing data | 16 (11.3%) | 7 (10.9%) | 9 (11.5%) | p = 0.20 |
|  | No | 37 (29.4%) | 20 (35.1%) | 17 (24.6%) |  |
|  | Yes | 89 (70.6%) | 37 (64.9%) | 52 (75.4%) |  |
| Urinary dysfunction | Missing data | 42 (29.6%) | 26 (40.6%) | 16 (20.5%) | p = 0.61 |
|  | No | 32 (32.0%) | 11 (28.9%) | 21 (33.9%) |  |
|  | Yes | 68 (68.0%) | 27 (71.1%) | 41 (66.1%) |  |
| NOH | Missing data | 23 (16.2%) | 14 (21.9%) | 9 (11.5%) | p = 0.83 |
|  | No | 37 (31.1%) | 15 (30.0%) | 22 (31.9%) |  |
|  | Yes | 82 (68.9%) | 35 (70.0%) | 47 (68.1%) |  |
| Photophobia | Missing data | 0 (0.0%) | 0 (0.0%) | 0 (0.0%) | p = 0.65 |
|  | No | 57 (40.1%) | 27 (42.2%) | 30 (38.5%) |  |
|  | Yes | 85 (59.9%) | 37 (57.8%) | 48 (61.5%) |  |
| Smell disorders | Missing data | 1 (0.7%) | 1 (1.6%) | 0 (0.0%) | p = 0.93 |
|  | No | 71 (50.4%) | 32 (50.8%) | 39 (50.0%) |  |
|  | Yes | 70 (49.6%) | 31 (49.2%) | 39 (50.0%) |  |
| Taste disorders | Missing data | 1 (0.7%) | 1 (1.6%) | 0 (0.0%) | p = 0.27 |
|  | No | 80 (56.7%) | 39 (61.9%) | 41 (52.6%) |  |
|  | Yes | 61 (43.3%) | 24 (38.1%) | 37 (47.4%) |  |

1. **Prevalence of autonomic and neurosensory disorders during the follow-up stratified by the intake of cholinesterase inhibitors**


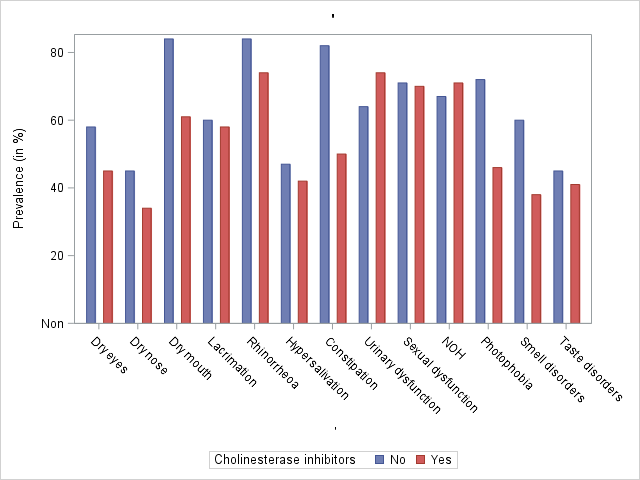


|  | | **Total (N=142)** | **No cholinesterase inhibitors (N=75)** | **Cholinesterase inhibitors (N=67)** | **p-value** |
| --- | --- | --- | --- | --- | --- |
| Dry eyes | Missing data | 11 (7.7%) | 6 (8.0%) | 5 (7.5%) | p = 0.14 |
|  | No | 63 (48.1%) | 29 (42.0%) | 34 (54.8%) |  |
|  | Yes | 68 (51.9%) | 40 (58.0%) | 28 (45.2%) |  |
| Dry nose | Missing data | 11 (7.7%) | 6 (8.0%) | 5 (7.5%) | p = 0.20 |
|  | No | 79 (60.3%) | 38 (55.1%) | 41 (66.1%) |  |
|  | Yes | 52 (39.7%) | 31 (44.9%) | 21 (33.9%) |  |
| Dry mouth | Missing data | 11 (7.7%) | 6 (8.0%) | 5 (7.5%) | **p = 0.003** |
|  | No | 35 (26.7%) | 11 (15.9%) | 24 (38.7%) |  |
|  | Yes | 96 (73.3%) | 58 (84.1%) | 38 (61.3%) |  |
| Lacrimation | Missing data | 10 (7.0%) | 5 (6.7%) | 5 (7.5%) | p = 0.82 |
|  | No | 54 (40.9%) | 28 (40.0%) | 26 (41.9%) |  |
|  | Yes | 78 (59.1%) | 42 (60.0%) | 36 (58.1%) |  |
| Rhinorrhoea | Missing data | 2 (1.4%) | 1 (1.3%) | 1 (1.5%) | p = 0.16 |
|  | No | 29 (20.7%) | 12 (16.2%) | 17 (25.8%) |  |
|  | Yes | 111 (79.3%) | 62 (83.8%) | 49 (74.2%) |  |
| Hypersalivation | Missing data | 6 (4.2%) | 3 (4.0%) | 3 (4.5%) | p = 0.56 |
|  | No | 75 (55.1%) | 38 (52.8%) | 37 (57.8%) |  |
|  | Yes | 61 (44.9%) | 34 (47.2%) | 27 (42.2%) |  |
| Constipation | Missing data | 5 (3.5%) | 2 (2.7%) | 3 (4.5%) | **p < 0.0001** |
|  | No | 45 (32.8%) | 13 (17.8%) | 32 (50.0%) |  |
|  | Yes | 92 (67.2%) | 60 (82.2%) | 32 (50.0%) |  |
| Sexual dysfunction | Missing data | 16 (11.3%) | 6 (8.0%) | 10 (14.9%) | p = 0.92 |
|  | No | 37 (29.4%) | 20 (29.0%) | 17 (29.8%) |  |
|  | Yes | 89 (70.6%) | 49 (71.0%) | 40 (70.2%) |  |
| Urinary dysfunction | Missing data | 42 (29.6%) | 17 (22.7%) | 25 (37.3%) | p = 0.29 |
|  | No | 32 (32.0%) | 21 (36.2%) | 11 (26.2%) |  |
|  | Yes | 68 (68.0%) | 37 (63.8%) | 31 (73.8%) |  |
| NOH | Missing data | 23 (16.2%) | 12 (16.0%) | 11 (16.4%) | p = 0.58 |
|  | No | 37 (31.1%) | 21 (33.3%) | 16 (28.6%) |  |
|  | Yes | 82 (68.9%) | 42 (66.7%) | 40 (71.4%) |  |
| Photophobia | Missing data | 0 (0.0%) | 0 (0.0%) | 0 (0.0%) | **p = 0.002** |
|  | No | 57 (40.1%) | 21 (28.0%) | 36 (53.7%) |  |
|  | Yes | 85 (59.9%) | 54 (72.0%) | 31 (46.3%) |  |
| Smell disorders | Missing data | 1 (0.7%) | 0 (0.0%) | 1 (1.5%) | **p = 0.009** |
|  | No | 71 (50.4%) | 30 (40.0%) | 41 (62.1%) |  |
|  | Yes | 70 (49.6%) | 45 (60.0%) | 25 (37.9%) |  |
| Taste disorders | Missing data | 1 (0.7%) | 0 (0.0%) | 1 (1.5%) | p = 0.60 |
|  | No | 80 (56.7%) | 41 (54.7%) | 39 (59.1%) |  |
|  | Yes | 61 (43.3%) | 34 (45.3%) | 27 (40.9%) |  |
