# Supplementary material for: Autonomic and neurosensory disorders in dementia with lewy bodies: prevalence and neural basis in the AlphaLewyMA cohort
Source: Alzheimers Res Ther. 2025 Dec 19;17:271. doi: 10.1186/s13195-025-01935-z (PMC12751402; doi:10.1186/s13195-025-01935-z)
Supplement: Supplementary file 4 — Supplementary Material 4 [file 13195_2025_1935_MOESM4_ESM.docx]

## Additional file 4: Fluctuating nature of AUD and NSD during follow-up

Each line represents a subject who was symptomatic for the AUD/NSD considered. Red and green boxes represent follow-up visits associated with the presence (red) or absence (green) of symptoms.

|  | **Median [Q1-Q3] of the ratio of visits marked by a change in AUD/NSD reporting (compared with the previous visit)** |
| --- | --- |
| **Dry eyes** | 0.29 [0.17 -0.33] |
| **Dry nose** | 0.29 [0.22 - 0.39] |
| **Dry mouth** | 0.25 [0.17 -0.33] |
| **Lacrimation** | 0.25 [0.17 -0.29] |
| **Rhinorrhoea** | 0.22 [0.13 -0.25] |
| **Hypersalivation** | 0.29 [0.17 -0.30] |
| **Constipation** | 0.22 [0.13 -0.29] |
| **Urinary dysfunction** | 0.20 [0 -0.25] |
| **Sexual dysfunction** | 0.25 [0 -0.33] |
| **Neurogenic orthostatic hypotension** | 0.33 [0.25 -0.40] |
| **Photophobia** | 0.25 [0.11 -0.33] |
| **Smell disorders** | 0.29 [0.15 -0.33] |
| **Taste disorders** | 0.22 [0.17 -0.33] |

AUD = autonomic disorders; DLB = dementia with Lewy bodies; NSD = neurosensory disorders; Q1-Q3 = 1st and 3rd quartiles
